# Diverse and flexible behavioral strategies arise in recurrent neural networks trained on multisensory decision making

**DOI:** 10.1371/journal.pcbi.1013559

**Published:** 2025-10-09

**Authors:** Thomas S. Wierda, Shirin Dora, Cyriel M. A. Pennartz, Jorge F. Mejias

**Affiliations:** 1 Cognitive and Systems Neuroscience Group, Swammerdam Institute for Life Sciences, University of Amsterdam, Amsterdam, The Netherlands; 2 Department of Computer Science, School of Science, Loughborough University, Loughborough, United Kingdom; RU Nijmegen Donders Institute: Radboud Universiteit Donders Institute for Brain Cognition and Behaviour, NETHERLANDS, KINGDOM OF THE

## Abstract

Behavioral variability across individuals leads to substantial performance differences during cognitive tasks, although its neuronal origin and mechanisms remain elusive. Here we use recurrent neural networks trained on a multisensory decision-making task to investigate inter-subject behavioral variability. By uniquely characterizing each network with a random synaptic-weights initialization, we observed a large variability in the level of accuracy, bias and decision speed across these networks, mimicking experimental observations in mice. Performance was generally improved when networks integrated multiple sensory modalities. Additionally, individual neurons developed modality-, choice- or mixed-selectivity, these preferences were different for excitatory and inhibitory neurons, and the concrete composition of each network reflected its preferred behavioral strategy: fast networks contained more choice- and mixed-selective units, while accurate networks had relatively less choice-selective units. External modulatory signals shifted the preferred behavioral strategies of networks, suggesting an explanation for the recently observed within-session strategy alternations in mice.

## Author summary

Differences in the performance of any given task between individual subjects, or within session for the same individual, are important but often ignored in behavioral studies. In this work, we use neuro-AI methods, particularly training of recurrent neural networks, to show that individual behavioral variability may arise simply from randomness within the structure of neural networks trained to perform a decision-making task. The different behavioral strategies are also reflected in the underlying activity properties once the neural networks have learned the task, establishing a testable link between neurophysiology and behavioral output. External modulation of these networks explains the alternating strategies observed at the individual level within a single session. This opens new avenues to use AI-based approaches to better understand inter-subject and inter-trial behavioral variability.

## Introduction

Individuals usually display a high level of variability in their cognitive abilities and goal-directed behavior [1,2], often exceeding what would be expected even from genetically identical subjects [3–5]. When performing cognitive tasks such as decision making [6–9] or multisensory integration [10–15], individuals have often been shown to develop separate choice strategies [16–20]. In the case of mice performing decision-making tasks, Pittaras and colleagues observed a broad distribution of behavioral strategies depending on the risk-aversiveness of the animals [17]. Rats performing context-dependent decision-making tasks display individually different behavioral strategies, even when their performance levels were similar [18]. Behavioral strategies are, furthermore, not an invariant quality for a given animal, as mice have been shown to alter their decision strategy within the same session [19,20]. While the mechanisms underlying different behavioral strategies are still unclear, there is evidence of behavioral strategies being reflected in neurobiological features [17], suggesting a connection between behavioral variability and neural and circuit heterogeneity, which is already known to play a beneficial role in neural processing and computations [21–25].

In spite of its prevalence and impact, this inter-subject behavioral variability is still treated in most studies as noise, and averaged out across subjects in behavioral and neuroscience research on both human and non-human animal studies [1,2]. In the absence of systematic behavioral datasets or standardized protocols to control for behavioral variability in rodents, computational models offer a practical way to explore the impact of behavioral variability in experiments [18]. Models of recurrent neural networks (RNNs) are particularly well suited for this task, since they can be easily trained to perform simple perceptual, cognitive and behavioral tasks mimicking the ones used in actual experiments [26–32]. Unfortunately, most computational studies also average over different RNN realizations to present cohesive results, and therefore the potential of RNNs to uncover the origin and role of inter-subject behavioral variability has been largely untapped.

In this work, we trained a large number of RNNs on a simplified version of a classical multisensory stimulus discrimination task [11,33] and explored the emergence of inter-subject variability in behavioral strategies within these networks. Each network was initialized with a unique set of synaptic weights before training, to reflect the natural variability present across subjects. The RNNs also included excitatory and inhibitory neurons (with a ratio of 4-to-1) to account for Dale’s law and enhance their biologically plausibility [28]. We found that training these initially heterogeneous RNNs under the same protocol led to a large variability in terms of reaction time, choice bias and accuracy of decisions, as reflected by their individual psychometric and chronometric curves. In addition, neurons within each network showed heterogeneous responses and could be classified as modality-selective, choice-selective, mixed-selective (i.e., modality and choice), hyper-selective, or silent [34]. There was a clear correlation between a network’s behavioral strategy and its neural composition: fast-deciding networks differed from slow ones in the proportion of choice-selective, mixed-selective and silent neurons, and accurate networks differed from inaccurate ones in the proportion of choice-selective units. The structure of these functional signatures was also different for excitatory and inhibitory neurons, suggesting differentiated roles between both classes [35]. Finally, we found that external modulatory currents were able to shift the preferred strategy of a given network, suggesting that neuromodulatory signals (such as acetylcholine or noradrenaline) or gating signals from other brain areas (such as thalamic nuclei) could be behind the within-session alternations in strategies observed in mice [19].

## Results

**Fig 1:**
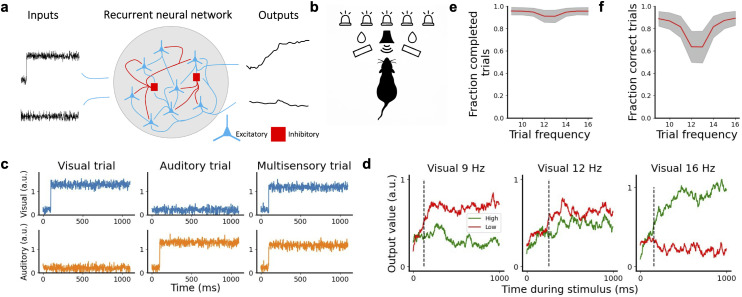
Recurrent neural network to model multisensory integration. **a** We considered 200 RNNs, each of them having 150 neurons (80% excitatory neurons and 20% inhibitory neurons), which received two streams of input and were trained to decide if the level of the input was above or below a threshold. The networks reported a choice by elevating the corresponding output variable. **b** Experimental task proposed by Raposo et al. and modeled in this work. In the original task, rats received visual flashes and/or auditory clicks and had to report whether the frequency was higher of lower than an experimenter-imposed threshold frequency. **c** Examples of input received by the model on visual (left), auditory (center) and multimodal (right) trials, via the visual (top) or auditory stream (bottom). **d** Examples of a network’s output variables throughout the trial on easy low (left), difficult (center), and easy high (right) trials. Dashed lines indicate the decision time of the trial. **e** Mean fraction of valid trials a network had per trial frequency, with the grey band indicating the s.e.m. across networks. **f** Mean fraction of trials with a correct decision for different trial frequencies.

We trained 200 RNNs (Fig 1a) on a simplified version of a standard multisensory integration task [11,33], in which the animal had to decide whether the rate of a series of sensory pulses was higher or lower than a known reference level (Fig 1b). The pulses corresponded to flashes for visual trials, clicks for auditory ones, and combined flashes-clicks for audiovisual ones. In our model, each network consisted of 150 rate-based neurons, of which 80% were excitatory and 20% inhibitory [28]. Each network received two streams of input (one corresponding to visual input and the other to auditory input), and two output variables were produced from the excitatory population, coding for the two choices the network could make (high vs low rate). Sensory input was modeled as noisy step currents (Fig 1c), whose amplitude represented visual and/or auditory pulse frequencies between 9 Hz and 16 Hz, with 12.5 Hz corresponding to the threshold between both options. On unimodal trials, the network received information for one modality, whereas on multimodal trials the network received congruent information about the frequency in both modalities (Fig 1c). The network made a categorical decision by raising the correct output variable while keeping the other low (Fig 1d). The stimulus was presented for 1 second after a fixation period of 100 ms in which the network had to keep both output variables low. A choice was recorded when the output variables were separated by more than 0.2 (given in arbitrary units for neural activity, with alternative values explored in S1 Fig), and a trial was considered invalid if a choice was registered before stimulus onset. On average, networks showed a high fraction (> 0.9) of valid trials after training on all frequencies, with a small dip in the number of valid trials around the threshold frequency (Fig 1e). After training, we generated 8192 trials per model with equal probability across all conditions. Networks were generally able to perform the task well, with a decrease in performance for difficult trials presenting a frequency close to the threshold frequency (Fig 1f, see Methods for more details).

### Networks display a significant variability in task performance

**Fig 2:**
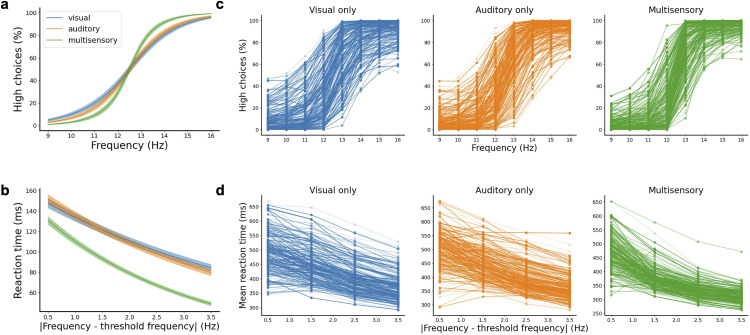
Networks show variability in task performance. **a** Psychometric functions averaged over all networks for visual (blue), auditory (orange) and multisensory (green) trials, where an improvement of multisensory over unisensory trials can be observed. Shaded bands represent the s.e.m. across networks. **b** Chronometric functions averaged over all networks on visual (blue), auditory (orange) and multisensory (green) trials; lower reaction times are observed for multisensory compared to unisensory trials. **c** Psychometric functions for individual networks, displaying a large variability across them. **d** Chronometric functions for individual networks, displaying a large variability across them.

To characterize the behavioral output of each network, we calculated its psychometric curves (i.e., fraction of times the network chose ‘high’ as output vs input frequency) and chronometric curves (time to reach a decision vs input frequency) after training. For psychometric curves, only trials in which a decision was made during stimulus presentation were included. The chronometric curves were calculated based on trials resulting in a correct choice only. On average, networks showed improvement in their performance (Fig 2a) and needed less time to form a decision (Fig 2b) on multimodal trials as compared to unimodal trials, replicating experimental findings [11,33]. Notice that all networks were trained with the same protocol, and their behavioral differences are a consequence of the unique initial conditions (i.e., randomized weights) of each network before training, reflecting innate or pre-training variability across subjects. Furthermore, the 200 networks showed great variability in both their psychometric functions (Fig 2c) and reaction times (Fig 2d). This indicates that different networks could potentially adopt different behavioral strategies to solve the task, such as a fast-but-inaccurate strategy, a slow-but-accurate strategy, a preferentially-choose-low strategy, etc. We then sought to explore whether this was indeed the case, and whether structural differences were underlying the different strategies adopted by networks.

### Multisensory trials lead to heterogeneous, but faster and more accurate responses

**Fig 3:**
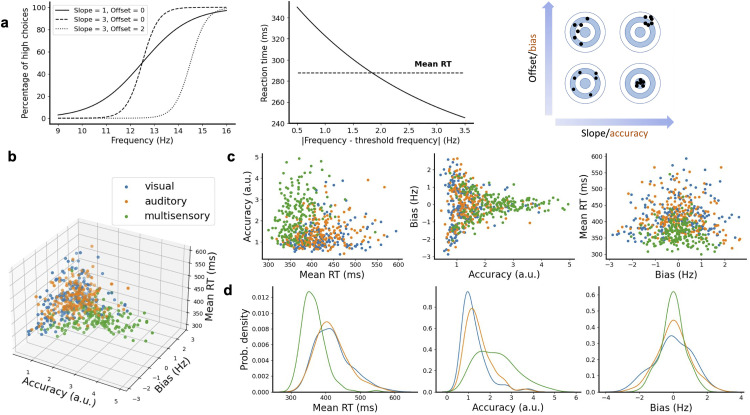
Multisensory trials lead to faster and more accurate network responses. **a** Sigmoidal (left panel) and exponential curves (middle panel) used to fit each network’s psychometric and chronometric data. The slope and frequency offset of the sigmoidal curves were used as fitting parameters and to estimate, respectively, the accuracy and choice bias of each network (right panel). The decision speed was estimated by computing the mean value of the reaction times for correct trials. **b** A 3D scatter plot reveals significant behavioral variability across all 200 networks and three conditions (visual, auditory and multisensory trials). **c** 2D projections of the 3D scatter plot for different pair of metrics. **d** The distribution of reaction times, accuracy and choice bias for visual, auditory and multisensory trials.

We used the psychometric and chronometric functions of the networks to map network performance to several behavioral metrics. For example, the slope parameter of the psychometric curve gives an indication of how well-defined the decision threshold of the network is (Fig 3a, left panel). A large slope value indicates that the network can confidently classify each input into one of the two outputs, whereas a small slope suggest that the network makes more mistakes in the classification of inputs of intermediate frequencies. We used this as a measure of network accuracy. The frequency offset parameter of the psychometric curve gives an indication of the frequency at which the network switches from low to high choices. We therefore used this as a measure of choice bias. To assess the decision speed of a network, we used the mean reaction time of all the network’s correct choices (Fig 3a, middle panel). The combination of accuracy and choice bias (Fig 3a, right panel) with the mean reaction time constitutes our full set of metrics to characterize a network’s behavioral strategy.

When mapped over the different trial modalities (visual, auditory and multisensory) we observed that networks showed a high variability in all metrics across the different modalities (Fig 3b). We also observed that some networks had a low accuracy with a high reaction time, whereas others had a high accuracy with a low reaction time. High accuracy with low reaction time was predominantly achieved on multisensory trials (green dots) rather than unimodal trials (orange/blue dots). We observed a positive correlation between the accuracy and the time it took to make a decision (S2 Fig), indicating a speed-accuracy trade-off [36–38].

To explore these relationships more precisely, 2D-projections of the 3D scatter plot were scrutinized, comparing pairs of metrics (Fig 3c). Low mean reaction times and high accuracy decisions were mostly achieved in multisensory integration trials, while unisensory trials led to slower and less accurate decisions (Fig 3c, left). Comparing the accuracy with the choice bias (Fig 3c, center) showed that the multisensory trials resulted in a wider range of accuracy values than those for unisensory trials, and that the more accurate a network was, the less biased they became. Comparing the bias with the mean reaction time (Fig 3c, right) again demonstrated that networks generally decided faster on multisensory trials, but not at the cost of choice bias. To further quantify these 2D-projections we calculated the distributions of the different metrics (Fig 3d). In multisensory trials the distribution of the mean reaction time was skewed more to the left than for either type of unimodal trial, with no major differences between unimodal trials (Fig 3d, left). The distribution of accuracy values is wider for multisensory trials as compared to unimodal trials, with approximately the same minimum value but a larger maximum value (Fig 3d, center). The distribution plot of choice bias showed that the networks achieve the lowest bias mostly on multisensory trials (Fig 3d, right). Fitting a more extended sigmoidal curve with lapse parameters, which account for shifts in percentage values of psychometric curves, gave similar results (S3 Fig and section A of the S1 Table).

### Network dynamics and units show heterogeneous responses and vary between strategies

**Fig 4:**
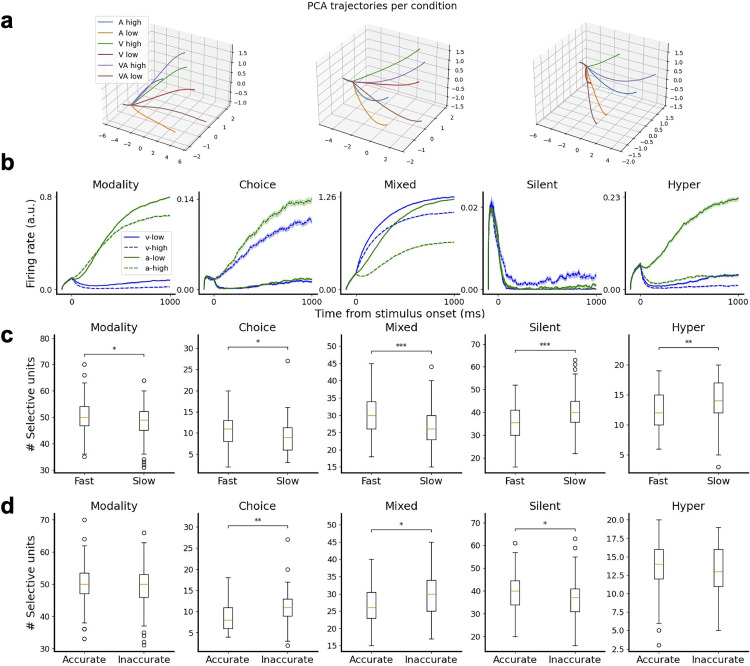
Network dynamics and units show heterogeneous responses and vary between strategies. **a** Principal component analysis decomposition of the dynamics of three example networks (left, middle and right panels), displaying the dynamical trajectories of overall network activity for different types of trials (coded by colors). **b** Example of network units showing modality selectivity, choice selectivity, mixed selectivity, silence during the stimulus and hyper-selectivity. **c** Number of selective units, across all five selectivity categories, for fast vs slow networks. **d** Same as **c**, but for accurate vs innacurate networks. Significance: * p < 0.05, ** p < 0.01, *** p < 0.001 two-tailed permutation test with n = 100,000 resamples and Holm-Bonferroni correction.

To explore how the internal neural dynamics of the different networks may give rise to varying behavioral strategies, we analyzed the dynamical trajectories of the neural activity of our RNNs using principal component analysis (PCA). Fig 4a shows, for three example networks, the average neural dynamics trajectories for different modality and choice conditions (e.g., auditory high-choice trials, audiovisual low-choice trials, etc). We see that all networks are able to clearly separate the dynamical trajectories, according to the specific conditions, from the moment that the integration process begins (the ramification point of all trajectories). The degree of separation per condition seems to differ across networks: for example, the network in the left discriminates more clearly between high and low frequency trials, regardless of the modality of the condition. The network shown in the middle panel, however, separates the trajectories more consistently by modality (visual versus auditory) and not separated as much based on choice. This suggests that each network performs the task by using unique neural dynamics trajectories. Such global dynamical trajectories may be the consequence of network-to-network differences down to the level of individual units. To explore this possibility, we turn now to the study of the activity of individual neurons in our networks.

Upon examining the dynamics of neurons within the networks, we observed that they showed heterogeneous responses to different unimodal trial conditions. We classified neurons based on their firing rate activity on correct unimodal trials and distinguished five different types of selectivity: (1) modality selectivity, (2) choice selectivity, (3) mixed selectivity (i.e., a combination of modality and choice selectivity [34]), (4) silent or very-low-activity neurons, and (5) hyper-selectivity (Fig 4b). To assess whether networks showing different strategies differed in their underlying physiological response properties and functional structure, we categorized the networks into groups based on their overall accuracy and mean reaction time irrespective of modality. A dichotomy between fast versus slow, and accurate versus inaccurate networks was adopted based on the average of the metrics across all networks. Next, the number of selective units of the different types was compared between the groups, where differences could suggest that these units play a role in the formation of a network’s strategy. Fast networks significantly differed from slow networks in all categories (Fig 4c), with the differences being highly significant for the case of mixed-selective neurons (more common in fast networks, p < 0.001) and silent neurons (more common in slow networks, p < 0.001). Accurate networks also displayed differences with respect to inaccurate ones (Fig 4d), with the most notable difference being that accurate networks have significantly less choice-selective neurons (p < 0.01) than inaccurate networks (see section B of the S1 Table for more details on the comparisons).

The absolute number of selective units was furthermore associated with the reaction time and accuracy of the networks (section C of the S1 Table). In particular, the number of choice- and mixed-selective units was negatively correlated with accuracy (r = -0.3, p < 0.01 and r = -0.29, p < 0.01 respectively) and and also with reaction time (r = -0.42, p < 0.001 and r = -0.45, p < 0.001 respectively), revealing speed-accuracy tradeoffs for these groups. Silent neurons displayed a positive correlation with both accuracy (r = 0.36, p < 0.001) and reaction time (r = 0.49, p < 0.001), therefore also supporting a speed-accuracy tradeoff but in the opposite direction.

### Inhibitory and excitatory populations differ in selectivity

A relevant question here is whether the distinction between excitatory and inhibitory neurons plays any functional role in the results found so far. A first attempt at exploring this issue is to ignore the Dale’s principle assumed until now in our model and allow any neuron to have positive and negative outgoing synapses. Simulations for this more artificial model show a similar spread of behavioral properties (S4 Fig and section D of the S1 Table), as well as proportions of selectivity units in line with those shown in Fig 4. However, this does not allow to disentangle the effects of excitatory and inhibitory contributions in the patterns observed between selectivity and behavior. To this aim, in this section we directly compare, in our original model satisfying Dale’s principle, the ratio between excitatory and inhibitory neurons within each selectivity group, as well as within different behavioral categories (i.e., fast, slow, accurate and inaccurate).

**Fig 5:**
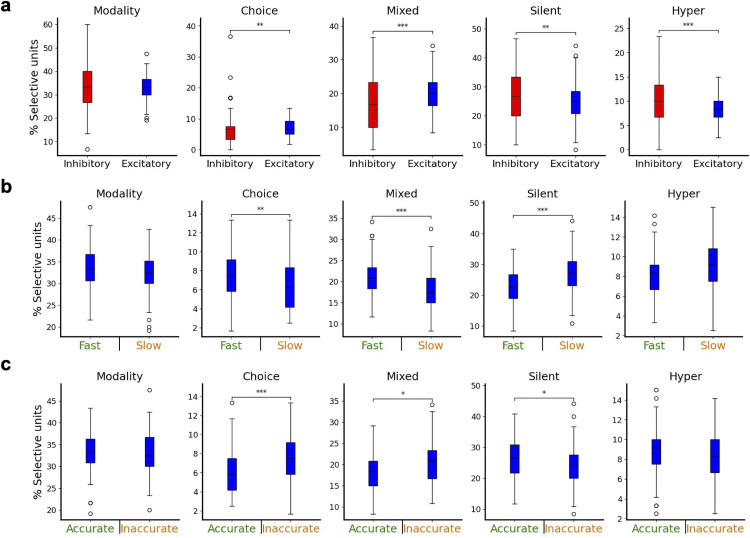
Inhibitory and excitatory populations differ in selectivity. **a** Comparison between the fraction of excitatory and inhibitory neurons found across all five selectivity types. **b** Fraction of excitatory neurons across all selectivity types for fast vs slow networks. **c** Same as **b**, but for accurate vs inaccurate networks. Significance: * p < 0.05, ** p < 0.01, *** p < 0.001 two-tailed permutation test with n = 100,000 resamples and Holm-Bonferroni correction.

On a population level, we observed that the ratio between excitatory and inhibitory neurons differs significantly (p < 0.001) for all selectivity groups except for modality (Fig 5a and section E of the S1 Table). In particular, choice- and mixed-selective units were predominantly excitatory, and silent and hyper-selective units were predominantly inhibitory. To further investigate whether the differences in strategies between networks could be attributed to differences between inhibitory and excitatory populations, and to different selectivity groups, we performed multiple comparisons between groups (section F of the S1 Table). When considering only excitatory neurons, this revealed that fast networks had significantly more choice- (p < 0.001) and mixed-selective (p < 0.001) neurons, but less silent (p < 0.001) neurons, than slow networks (Fig 5b). Likewise, we found that accurate networks predominantly differ from inaccurate ones in that they had significantly less (p < 0.001) choice-selective units (Fig 5c), indicating that having a large fraction of choice-selective neurons does not necessarily lead to better performance.

Overall, these results identify choice-, mixed-selective and silent neurons as the major factor differentiating fast vs slow networks, and choice-selective neurons for the case of accurate vs inaccurate ones. Speed-accuracy tradeoffs are likewise found for all three groups (Fig 5b and 5c), in line with our results above (Fig 4c and 4d and section C of the S1 Table). No significant differences were found between the groups in the inhibitory populations (S5 Fig), suggesting that differences observed in Fig 4c,d are mainly driven by those between excitatory groups.

### Modulatory currents alter the adopted behavioral strategies

**Fig 6:**
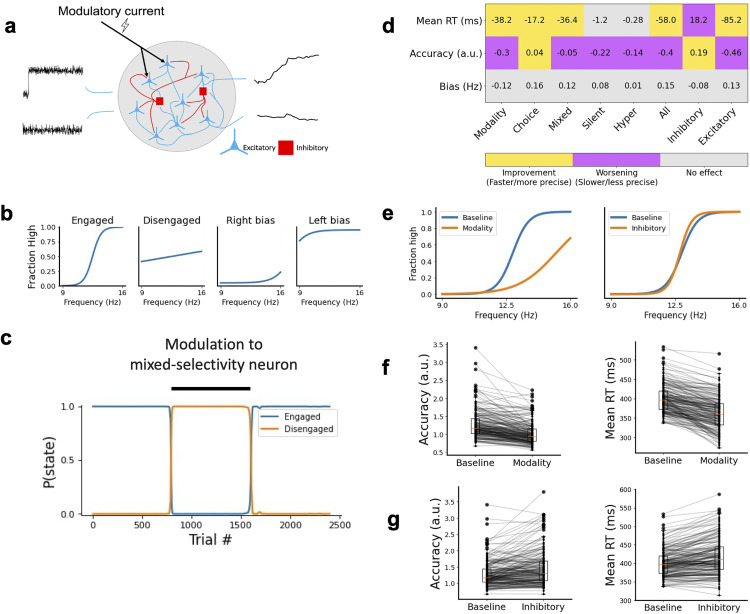
Modulatory currents can influence individual network strategies. **a** We applied a continuous modulatory current to different neuron subsets within a network to assess whether external modulation could influence individual behavioral strategies. **b** Potential effects of external modulation included networks becoming more engaged, disengaged, biased, and influence their reaction times. **c** Temporal evolution of behavioral state probabilities as revealed by a GLM-HMM approach. Externally modulating mixed-selective neurons leads to loss of accuracy and a transition from an engaged to a disengaged state. **d** Behavioral changes (as changes in reaction times, accuracy and bias) observed when the modulatory input targeted different groups (modality-selective, choice-selective, etc). Significant improvements (i.e., faster or more accurate decisions) are displayed in yellow blocks, worsening in magenta, and non-significant changes in grey. **e** Example of an individual network’s change in psychometric curves on the application of a modulatory current to its modality-selective (left) and its inhibitory (right) neurons. **f** Effect of modulatory input targeting modality-selective neurons in the accuracy (left) and reaction times (right) across all networks. **g** Effect of modulatory input targeting all inhibitory neurons in the accuracy (left) and reaction time (right) across all networks. The effects in panels **f** and **g** are opposite to each other, and may serve to rebalance speed-accuracy trade-offs in the network’s behavior.

To explore whether our model was able to explain sudden changes in a subject’s behavioral strategy, as experimentally reported [19,20], we studied the effect of adding an extra modulatory (constant) current to the network (Fig 6a), which is aimed to reflect internal gating or neuromodulation. Across eight different experiments, eight different groups of neurons received this modulatory current throughout the entire trial duration. The eight groups that received the current were: all five different types of selective units, modulated one at a time (1–5), all neurons (6), inhibitory neurons only (7) and excitatory neurons only (8). The application of a modulatory current could alter the accuracy, bias and decision speed of the network, or in the terminology of Ashwood et al. [19], could lead to a network becoming more engaged, disengaged, biased or exhibiting changes in reaction times (Fig 6b). Following Ashwood and colleagues, we use GLM-HMM to estimate the behavioral state of networks across trials. As suspected, modulatory currents are able to alter the default behavioral strategies of a network: for example, we can transiently drive a engaged-by-default network towards a disengaged state by injecting an external modulatory current to its mixed-selective population (Fig 6c). Similar conclusions can be obtained when we evaluate the behavioral strategies via psychometric and chronometric curves as before, which we did to perform an exhaustive exploration for external modulation across all eight groups defined above (Fig 6d). We observe then that reaction times decreased (p < 0.001) when the modulatory current targeted modality-selective, choice-selective, mixed-selective, excitatory, or all neurons, while they increased (p < 0.001) with input targeting inhibitory neurons (Fig 6d and section G of the S1 Table). On the other hand, accuracy increased when the modulatory input targeted choice-selective (p < 0.05) and inhibitory (p < 0.001) neurons, and decreased (p < 0.05) when any of the other groups were targeted. This led to two interesting traits: (i) speed-accuracy trade-offs were observed when the modulatory signal targeted either modality-selective, mixed-selective, or any structural-based group (excitatory, inhibitory, or all neurons), and (ii) modulating choice-selective neurons led to improvements in both accuracy and decision speed. The modulatory current did not cause any significant shift in the bias of the models. Although on an individual network level we found changes in bias levels (Fig 6e, left), the direction of the shift differed a lot between the networks resulting in no significant average shift to either direction. Similar results were found when considering other criteria for neural selectivity, such as one based on ROC curves [33,35] (S6 Fig and section H of the S1 Table).

An example network, shown in Fig 6e, illustrates the opposing example of the effects that a modulatory current has on the network’s accuracy when targeting a group. In the first example, which considers an input targeting modality-selective neurons, the modulatory current lowers the accuracy (and introduces a right-shift bias) with respect to the baseline or control case. In the second example, in which the input targets all inhibitory neurons, the current increases the accuracy of the network. Changes in accuracy and reaction times across all networks for these two modulation examples are respectively shown in Fig 6f and 6g. These examples demonstrate that externally modulating modality-selective neurons or inhibitory neurons lead to speed-accuracy trade-offs of opposing signs, and therefore can be effectively used to alter the behavioral strategies of a network. To further explore the role not only of modulation, but also inactivation of neurons in the decision task, we performed a lesion experiment of the all groups of neurons (S7 Fig and section I of the S1 Table). This revealed that inactivating silent neurons led to faster and more accurate networks (presumably due to the reduction in noise caused by the weak activity of these neurons), while lesioning hyper-selective neurons led to an increase in accuracy. It also confirmed the relevance of choice and mixed-selective neurons in defining the network’s behavioral strategies.

While lesioning silent and hyper-selective neurons has opposite effects to their excitatory modulation (which leads to decreases in accuracy), as one might expect, this is not tru for all cell groups. Notably, excitatory modulation of choice-selective cells slightly increases the accuracy and reduces the reaction times, but lesioning those cell also leads to shorter reaction times. While contradictory on a first read, this can be explained if we assume that choice-selective neurons optimally contribute to having longer accummulation times in the network. Lesioning these cells would naturally lead to shorter reaction times, but an overexcitation driven by external modulation can also push them out of their optimal working point, leading to faster ramp-ups and shorter reaction times. Therefore, the effect of lesions and modulations in selectivity groups can lead to complex and nonlinear results.

## Discussion

In this work, we used a computational formalism based on RNNs to show that the natural behavioral variability observed in animals can be explained by initial configurational differences across brain networks undergoing the same training protocol. This behavioral variability reflects different strategies adopted by networks, including choice biases and different levels of speed-accuracy trade-offs [36–38], and can be externally altered via external modulatory currents [19]. Furthermore, we showed that networks tend to choose more accurately and faster in multimodal rather than unimodal trials, highlighting the importance of multisensory integration mechanisms in perception and in strong agreement with experimental evidence and computational frameworks [10,12–15,39–41]. Adopting a multisensory decision-making task was an optimal choice for this study, due to the task incorporating not only choice but also modality properties. We would however expect to find similar variability of behavioral strategies in other complex cognitive and behavioral tasks, as long as it can be rooted on similar physiological and functional heterogeneity principles.

### The link between neural selectivity and behavioral strategies

Trained networks developed heterogeneous single-unit responses to different trial conditions, revealing interesting relationships between different selective units and adopted strategies. Fast networks had more choice- and mixed-selective units and less silent neurons than slow networks. Accurate networks, on the other hand, had significantly less choice-selective neurons compared to inaccurate ones. Combining these findings suggests that an increased number of choice-selective neurons could lead to adopting a fast-but-inaccurate strategy, whereas the number of silent and mixed-selective units play a more important role for controlling speed of different decision-making strategies without affecting its accuracy (or with weaker speed-accuracy tradeoffs). Networks in which modality-, choice- and mixed-selective neurons are more balanced would therefore align with a slow-but-accurate behavioral strategy. Even though we observed differences in bias on an individual network level, there was not a systematic bias based on the number and type of selective units. See however our discussion on size effects and quantitative comparisons, particularly important for a computational study like ours, in the section ‘Statistical analyses’ of the Methods section.

The association between neural selectivity and behavioral strategies was not the same for excitatory and inhibitory neurons, suggesting that both cell types play different roles in task computations. Choice- and mixed-selective units were more commonly excitatory, while inhibitory neurons usually categorized as silent or hyper-selective units. Both excitatory and inhibitory neurons displayed similar levels of modality selectivity [15]. When considering networks with specific behavioral strategies, we observed that the high number of choice- and mixed-selective units and the low number of silent neurons in fast-decision networks (respect to slow-decision ones) could be attributed to differences in the behavioral strategy preferences of excitatory neurons. The same holds for the case of accurate and inaccurate networks, where again the significant difference in choice-selective units seemed to result from a difference in the excitatory population rather than the inhibitory population. This could imply that networks adopt different accuracy or speed strategies based on the percentage of excitatory selective units, and that inhibitory neurons support this differentiation by an internal fine-tuning which does not require a change in the total fraction of inhibitory selective units, or by regulating the stability and competition of those circuits [35,42]. Future RNN work adapting models with, for example, more diverse inhibitory types [43,44] should be able to uncover more differentiated roles.

We chose a firing rate-based approach for classifying selective units in contrast to other possible options such as a receiver-operator characteristic (ROC) approach [33,35]. We chose this approach as the rate-based framework allows for classifying more types of units (e.g., silent and hyper-selective neurons) as compared to the ROC approach (S6 Fig). We also opted to consider the different selectivity categories as exclusive (i.e., a mixed-selective neuron is not counted as also a choice-selective one); this explains the lower percentage of choice selective units as compared to other studies [33,35,45].

### Within-subject modulation of behavioral strategies

We were able to influence a network’s strategy by adding a modulatory current targeting specific groups of neurons. Signals from other brain regions, both cortical and subcortical, have been suggested to play gating or modulatory roles in cognitive functions [46–49], albeit their specific effects on RNNs performing complex neural computations like the ones considered here have not yet been studied in depth. Targeting different selective units resulted in different behavioral adaptations, with networks becoming faster, slower, or unchanged depending on which neuron groups were targeted. All types of possible change were also observed in terms of accuracy. As has been shown in recent work [19,20], animals tend to alternate strategies during experimental sessions, and our results indicate that this could be caused by varying levels of external input from, e.g., different brain areas targeting different sub-networks of neurons. Interestingly, targeting inhibitory neurons was the only manipulation in which networks generally became slower and more accurate, whereas networks become faster and less accurate upon the application of a modulatory current to most other cell groups. Targeting choice-selective neurons was the only condition in which the network became both faster and more accurate. For silent neurons it appeared that their number, rather than their firing-rate, influenced the network’s speed since we found that (i) fast networks have significantly less silent neurons compared to slow networks, but (ii) applying a modulatory current to silent neurons does not affect reaction times. Interestingly, forcing silent neurons to decrease their activity from spontaneous levels to zero improved network accuracy and reaction times (S7 Fig), in contrast to applying the modulatory current to silent neurons, which led to more inaccurate networks. This suggests that the low firing rate of silent neurons acts as a noise-control parameter in the system.

An important step in future studies will be to test our predictions for a wider set of perceptual and cognitive tasks, including those related to working memory, decision making and cognitive control [28,29]. These comparisons might not be, however, straightforward to consider, since several categories of neural selectivity might be specific for particular tasks (for example, modality-selective neurons are only relevant in multisensory tasks, when more than one modality is available). It is possible that the particular rules observed might depend on the categorization adopted for cellular selectivity. On the other hand, other categories are more robust across multiple tasks (for example, choice-selective neurons) and the influence of these groups might be more consistent across tasks.

Overall, our study suggests that the often-overlooked inter-subject behavioral variability contains useful information and reflects individual-specific strategies which may emerge from initial differences in neural networks under the same training protocol. These strategies are flexible (i.e., can be modulated by internal brain signals) and are reflected in specific neural signatures (i.e., selectivity properties). We consider that our RNN approach will allow to explore the origins and implications of cognitive and behavioral flexibility.

## Methods

All model development, training and data analysis was done using Python 3.9 and libraries including PyTorch, NumPy, matplotlib, SciPy and Sklearn.

### RNN model description

We trained 200 Recurrent neural networks (RNNs) based on earlier work [26,28]. The RNNs receive time-varying inputs and produce two outputs coding for the network’s decision (Fig 1a). The RNNs are described by discretized equations (or, equivalently, by differential equations whose discretization) of the form


$$\begin{array}{c} x_{t}=(\text{1}-\alpha )x_{t-1}+\alpha \left(W^{𝐫𝐞𝐜}r_{t-1}+b_{rec}+W^{𝐢𝐧}u_{t}+b_{inp}\right)+\sqrt{\text{2}\alpha \sigma_{\text{rec}}^{\text{2}}}N(\text{0},\text{1}), \end{array}$$ (1)

$$r_{t}={\left[x_{t}\right]}_{+},$$ (2)

$$z_{t}=W^{𝐨𝐮𝐭}r_{t_{𝐞𝐱𝐜}}+{𝐛}_{𝐨𝐮𝐭},$$ (3)

where α = Δt/τ and τ describes the time constant of the network. In these equations, $x_{t}$ is a vector describing the voltage of the neurons in the network at time t, and $r_{t}$ describes the firing rates of the neurons at the same time, which is obtained after applying a rectified linear (ReLU) nonlinearity to the voltage, $\left[x{]}_{+}=max(x,\text{0}\right)$. The neuron voltage decays with a factor of α at each timestep and receives the firing rates of the previous timestep $r_{t-1}$ via the matrix $W^{\text{rec}}$. At every timestep, the network also receives a time-varying input $u_{t}$ via matrix $W^{\text{in}}$. The output variables $z_{t}$ are obtained through $W^{\text{out}}$, which are given randomly fixed but positive values. Note that we only read out from the firing rates of the excitatory populations as this readout can be seen as a long-range projection onto other areas of the brain, which usually involves excitatory synapses. Noise is drawn from a normal distribution N(0,1) with zero mean and unit variance was drawn at every timestep and scaled accordingly. Additional constant terms $b_{rec},\;b_{inp},\;b_{out}$ describe the influence of other brain areas in the recurrent, input and output connections respectively. Each network consisted of 150 neurons of which 80% (120) was excitatory and 20% (30) was inhibitory, following Dale’s law [28]. An alternative description of our system can be given by a set of differential equations whose Euler-discretization aligns with the equations above.

The weights and biases of the input and recurrent computations were randomly initialized from a uniform distribution $U\left(-\sqrt{k},\sqrt{k}\right)$ with $k=\frac{\text{1}}{\#input\;features}$, where #input features is 5 and 150 for the input and recurrent units respectively. For the output weights and biases we used k = 0.01. Based on trial-and-error, we multiplied the initial weights of the inhibitory units in the recurrent matrix with 6 to improve convergence.

The details of the task specific input will be discussed in the next section, but the networks received an input of the form


$$u_{t}={\left[u^{0}+u_{t}^{task}+\frac{\text{1}}{\alpha }\sqrt{2\alpha \sigma_{\text{in}}^{\text{2}}}𝐍\left(0,1\right)\right]}_{+},$$ (4)

where $u^{0}$ represents a baseline input, $u_{t}^{task}$ the task specific input at timestep t, and noise was drawn from a normal distribution N(0,1) with zero mean and unit variance at every timestep and scaled accordingly. To ensure that the inputs are non-negative a rectified linear (ReLU) nonlinearity is applied.

### Task description

We have trained the RNNs on a multisensory integration task similar to the task described in Raposo and colleagues’ experimental work [11,33]. In this task, a rat sat in front of a monitor with a speaker which showed visual flashes and presented auditory clicks respectively (Fig 1b). The flashes and clicks were presented at different frequencies between 9 Hz and 16 Hz, and the animal needed to decide if the frequency was higher or lower than an experimenter-imposed threshold. Rats reported their decision by moving to one of two choice ports, where one port rewarded a decision for ‘higher-than-threshold’ and the other for ‘lower-than-threshold’.

We implemented a computational version of this task [28]. The networks received inputs coding for visual and auditory frequencies ranging between 9 Hz and 16 Hz. On unimodal trials, the network just received visual or auditory information, whereas on multimodal trials the network received both visual and auditory information about the frequency. When both modalities were presented, they were congruent. The network needed to decide if the frequency presented is higher or lower than the average frequency, or 12.5 Hz. The stimulus was presented for 1 second after a fixation period of 100 ms in which no stimulus was presented. The network reported a decision by raising one of the output variables while keeping the other output variable low and could report this at any time step during the trial. We registered a decision when the difference between the two output variables was larger than 0.2. A difference of 0.2 is chosen to mark a decision because a larger value increased the number of trials in which no decision is made, whereas a threshold lower than 0.2 increased the number of trials in which a decision is already made before the onset of the stimulus (S1 Fig). We both positively- and negatively tuned the inputs in order to improve the training [28]. After the models were trained, we generated 8192 trials, where the modality and the frequency were randomly drawn with equal probability across all conditions. For each trial we saved the network’s output and firing rates at every timestep.

### RNN training

The RNNS were trained to minimize the difference between their output variables ***z*** and target values ***T***. During the fixation period of the task, both output variables were trained to maintain a low value of 0.2. During the stimulus, the model should raise the correct output variable to a value of 1, while maintaining the other variable at the low value of 0.2. A training batch consisted of $N_{trials}=\text{2}\text{0}$ trials which were randomly generated at every epoch. After the random initialization of the networks, only the weights of $W^{\text{rec}}$ and the offsets $b_{rec},\;b_{inp},\;{𝐛}_{𝐨𝐮𝐭}$ were trained to stimulate the network to use the recurrent connections, rather than the input- and output connections, to solve the task. The weights and biases were trained using back-propagation through time (BPTT) to minimize the loss function


$$\begin{array}{c} L=\frac{\text{1}}{N_{\text{trial}}}\frac{\text{1}}{N_{\text{time}}}\frac{\text{1}}{N_{\text{out}}}\sum_{i=\text{1}}^{N_{\text{trial}}}\;\sum_{t=\text{1}}^{N_{\text{time}}}\;\sum_{o=\text{1}}^{N_{\text{out}}}\;M_{i,t}{\left(T_{i,t,o}-z_{i,t,o}\right)}^{\text{2}}+\;\lambda \sum_{n=\text{1}}^{{\text{N}}_{\text{params}}}\;x_{i,t,n}^{\text{2}} \end{array}$$ (5)

where $M_{i,t}$ is used as a mask for certain timepoints by using a value of 0 for timepoints that need to be excluded from the loss function and λ is the term that controls the L2-regularization to stimulate the networks to maintain small parameter values. The first 200 ms after stimulus onset were excluded from the loss function during training to avoid punishing the model for not directly making a decision and take into account biologically plausible processing times [26,28,31]. We performed the BPTT and parameter updates using stochastic gradient descent with the use of PyTorch’s SGD optimizer with a learning rate of 0.01 and MSEloss(), where the L2-regularization was implemented using the weight_decay() parameter of the SGD optimizer with a value of 0.1. In order to satisfy Dale’s law, excitatory weights that became negative or inhibitory weights that became positive during the updating step were reset to zero immediately after [28].

The networks were trained until they reached sufficient task performance, with a maximum of 10,000 epochs. If a model did not reach sufficient task performance within 10,000 epochs, or 200,000 trials, the training was terminated and model was not used for experimentation. Sufficient task performance was defined by two conditions: (1) at least 90% of trials was valid (no decision made before stimulus onset) and (2) the network made a correct choice in at least 80% of all valid trials. To assess network’s task performance during training we generated a validation batch of $N_{trials}=\text{1}\text{0}\text{2}\text{4}$ trials every 500 epochs and measured network performance. An overview of the training parameters is shown in Table 1.

**Table 1:** Parameter overview used for training and network design. All networks were trained with the parameters that are listed below. After random initialization, networks developed different structures following the same training procedure.

Parameter	Symbol	Value
Learning rate	η	0.01
Weight decay	λ	0.1
Recurrent noise	$\sigma_{rec}$	0.15
Input noise	$\sigma_{in}$	0.01
Baseline input	$u^{0}$	0.2
Time constant	τ	100
Timestep training	$\Delta t_{train}$	20
Timestep testing	$\Delta t_{test}$	2
Validation size training	$N_{trials}$	1024
Test size	$N_{trials}$	8192
Gradient minibatch size	$N_{trials}$	20
Maximum training epochs	$E_{max}$	10000
Number of neurons	$N_{neurons}$	150

We used Monte-Carlo cross-validation with samples generated using a simulation of the paradigm, enabling us to generate as many samples as required for both training and testing. For such scenarios, Monte-Carlo cross-validation is more suitable than K-Fold cross-validation as the final results are less affected by specific train/test splits.

### Performance metrics and strategy mapping

For each network we computed the psychometric function based on trials in which a decision was made during the stimulus presentation. Trials in which no decision was made, or a decision was made before the stimulus onset, were excluded from the analysis. For each frequency we computed the fraction of trials in which the network reported that the frequency was higher than 12.5 Hz and fit a sigmoidal function of the form


$$f(x)=\frac{\text{1}}{\text{1}+e^{-a(x-b)}},$$ (6)

where a is a slope parameter of the sigmoid and b the bias, with the use of the *curve_fit()* function from SciPy. We fitted this function to the difference of the frequency and the threshold of 12.5 Hz, resulting in a range of -3.5 Hz to 3.5 Hz centered around 0. We took the a of the fitted function as a measure of the accuracy of a network, as a higher a indicates a more abrupt change in choice from ‘lower-than-threshold’ to ‘higher-than-threshold’ frequency (Fig 3a, left). We took the b of the fitted function as a measure of bias, as a larger b indicates that a model shifts its sigmoidal curve to the right, hence changes from a ‘lower-than-threshold’ to ‘higher-than-threshold’ choice at a larger frequency than the 12.5 Hz (Fig 3a, left). The sigmoidal curve was fitted both per modality and across modalities. The former is done to show how models performed on unimodal versus multimodal trials in terms of accuracy and choice bias, whereas the latter is done to assess how accurate and biased a network is in general, which is used to investigate the different strategies that models adopt.

Alternatively, we have fitted a sigmoidal function with lapse parameters of the form


$$f(x)=\left(\text{1}-\left(\gamma_{r}+\;\gamma_{\lambda }\right)\right)\frac{\text{1}}{\text{1}+e^{-a(x-b)}}+\;\gamma_{r}$$ (7)

where $\gamma_{r}$ and $\gamma_{\lambda }$ account for guesses and lapses at the lowest and highest frequencies respectively. Lapses are defined as the fraction of ‘high’ choices at the lowest possible frequency, or the fraction of ‘low’ choices at the highest possible frequency [19,50] (S3c Fig).

For each network we also computed the chronometric function based on all correct trials. We calculated the average reaction time of the networks for different distances to the threshold frequency of 12.5 Hz (Fig 3a, center). We calculated this per modality to investigate the differences in reaction time between unimodal and multimodal trials. We took the average reaction time across all correct trials as a measure of how fast a network is in general, which is used to investigate the different strategies that models adopt. In order to rescale the calculated decision times to milliseconds, we multiplied our decision time, which is just the index of the time step, with Δt. To account for possible processing time of sensory inputs and motor outputs that are needed for decision making in a real biological system, we added a constant value of 200 ms to the obtained reaction times in order to report more biologically plausible reaction times.

To map these behavioral metrics to strategies, we labeled a network as fast if the network has a reaction time that is less than the average reaction time of all networks, and slow otherwise. We labeled a network as accurate if the slope of the fitted sigmoidal curve is larger than the average slope of all networks, and inaccurate otherwise.

### Neural dynamics analysis

We used principal component analysis (PCA) to analyse the dynamics of the network activity. To this aim, we considered the recordings of the firing rates of the neurons in the recurrent layer for each network. Only trials with correct responses were included in the analysis. To reduce the dimensionality of the hidden states, we first reshaped the data from [trials, time, number of neurons] to [trials * time, number of neurons] and applied PCA with three components to the full set of hidden activations. The reduced data was reshaped back to its original form, allowing us to compute average hidden-state trajectories over time per condition. Conditions were defined based on stimulus modality (visual, auditory, or audiovisual) and stimulus strength (low/high relative to a center frequency of 12.5 Hz).

This method allows us to qualitatively assess how the RNN’s hidden dynamics encodes task-relevant information and how separable those dynamics are across conditions.

### Measuring unit selectivity

Based on the firing rates of the units on correct trials [33] we assessed the selectivity of the neurons. Before the neurons were classified, the average firing rate across trial conditions was calculated. The trial conditions of interest were ‘auditory-high’, ‘auditory-low’, ‘visual-high’ and ‘visual-low’. The multimodal trials were excluded for this analysis as it would be ambiguous to attribute selectivity when two conditions were presented simultaneously. We defined five selectivity types (Fig 4b):

- **Modality:** when a neuron fires most on either auditory or visual trials irrespective of the frequency of the trial. For example, when a neuron fires more on auditory trials with a high and low frequency as compared to visual trials with either frequency, it is classified as modality-selective.
- **Choice:** when a neuron fires more for high- or low-frequency trials as compared to the opposite. For example, when a neuron fires more for auditory- and visual trials with a high frequency compared to auditory- and visual trials with a low frequency. Note that this is identical to firing more for a specific choice (high or low) since we only looked at correct trials.
- **Mixed:** when a neuron is both modality- or choice-selective. We do this by first classifying the neuron into one type (modality or choice) and then testing for the other one. In case of an initially-classified-as modality-selective neuron, we define this by looking if the firing rates also show separation based on high- versus low-frequency trials. This is done by taking the average of the firing rates for the high-frequency trials and separately the average of the firing rates for the low-frequency trials and measuring if the difference between those two is larger than the difference between the 2^nd^ and 3^rd^ largest firing rate conditions. In case of an initially-classified-as choice-selective neuron, this procedure is similar but the difference between the average firing rates of visual versus the average firing rates of auditory trials is compared against the difference between the 2^nd^ and 3^rd^ largest firing rate conditions.
- **Silent:** when a neuron fires less during the stimulus than the maximum firing rate during the fixation period for all unimodal trial types.
- **Hyper-selectivity:** when a neuron fires less during the stimulus than the maximum firing rate during the fixation period for three out of four unimodal trial types, but fires more for one, e.g., auditory high-frequency trials only.

We used the average firing rate over the last 20 timepoints, or 40 ms, of the trials to assess a neuron’s selectivity.

Alternatively, we computed the receiver operating characteristic [33,35] (ROC) and the area under this curve (AUC_ROC_) using the using roc_curve() and auc() functions from Sklearn. The same timepoints as in our original approach were used to determine the selectivities of the neurons. A neuron with no selectivity for modality or choice would have an AUC_ROC_ of 0.5. We determined if a neuron was selective by comparing the AUC_ROC_ of the neuron against the AUC_ROC_ of a shuffled distribution and marked a neuron selective if the AUC_ROC_ was in the lowest or highest 2.5 percentile of this shuffled distribution. In case of choice selectivity, the AUC_ROC_ was calculated based on the network’s choice, and for the shuffled distribution the choices were shuffled for 1000 times, while keeping the network’s activity the same. In case of modality selectivity, the trials were first split in trials ending in leftward and rightward choices. Then for each group, we checked how well the neuron’s activity predicts the trial modality (if it was visual or auditory). If in either the leftward or rightward trials the AUC_ROC_ differed significantly from a shuffled distribution in which the trial modalities are randomly shuffled, the neuron is marked as modality selective. Neurons that are marked both choice- and modality selective are labeled mixed selective.

### Adding modulatory current to selective units

To investigate to what extent we could change a network’s strategy, we added a modulatory current to specific neurons of each network during the trial, which experimentally could be achieved using optogenetics. In total 8 different experiments were performed targeting different sets of neurons. The 8 sets that were targeted were (1) modality selective neurons, (2) choice selective neurons, (3) mixed selective neurons, (4) silent neurons, (5) hyper-selective neurons, (6) all neurons, (7) inhibitory neurons, (8) excitatory neurons. We modulated the system by adding a constant bias current with noise to the targeted neurons, changing the equation of the current of the targeted neurons to


$${𝐱}_{t}=(\text{1}-\alpha ){𝐱}_{t-\text{1}}+\alpha \left(W^{rec}{𝐫}_{t-\text{1}}+{𝐛}_{\text{rec}}+W^{in}{𝐮}_{t}+{𝐛}_{\text{inp}}+{𝐛}_{\text{per}}\right)+\sqrt{\text{2}\alpha \sigma_{\text{per}}^{\text{2}}}𝐍(\text{0},\text{1})+\sqrt{\text{2}\alpha \sigma_{\text{rec}}^{\text{2}}}𝐍(\text{0},\text{1})$$ (8)

where a constant bias ${𝐛}_{per}$ of 0.2 is added and random noise sampled at every timestep with $\sigma_{per}=\text{0}.\text{0}\text{1}$. We generated $N_{trials}=\text{2}\text{0}\text{4}\text{8}$ trials per modulatory setup and added a baseline experiment of the same number of trials with no modulatory current against which the results of the modulatory currents were compared in terms of psychometric- and chronometric function. We added the modulatory current throughout the entire trial duration.

### Inferring behavioral strategies

We used a Hidden Markov model, particularly a GLM-HMM, to study alternating behavioral strategies in our networks (Fig 6c). We followed the methodology of Ashwood et al., therefore we only describe it briefly here. The goal of the approach is to infer the transition matrix $A\;\epsilon \;R^{K\times K}$, the set of weights influencing choice in each state ${\left\{w_{k}\right\}}_{k=\text{1}}^{K}$, with $w_{k}\;\epsilon \;R^{M}$, and the initial state distribution $\pi \;\epsilon \;R^{K}$. We fit this set of parameters, which we collectively denote as Θ, to choice data using maximum a posteriori estimation. In practice, this was implemented via the EM algorithm, which is described elsewhere.

### Lesion experiment

To investigate the role of silent neurons in the network, we performed an additional experiment where we effectively lesioned (or inactivate) different selectivity groups of neurons from the network. Note that, for the particular case of silent neurons, our definition allows for neurons that are marked silent to not be completely silent but rather have a firing rate lower than in the fixation period. To see if a selectivity group has any function, we perform an experiment in which we set the firing rates of those neurons to exactly zero, effectively lesioning them from the network. We compare the results of those lesioned networks against their baseline performance in terms of psychometric and chronometric function by generating $N_{trials}=\text{2}\text{0}\text{4}\text{8}$ trials.

### Statistical analyses

We used permutation tests of the difference in means with n = 100,000 resamples to assess if there were significant differences between groups (i.e., modality, choice, mixed, excitatory, inhibitory, etc). In case of comparisons between groups assigned to different strategies, we used the independent permutation test of SciPy which tests the null hypothesis that all observations are sampled from the same underlying distribution. For the experiments in which we apply a modulatory current to the networks, we used the paired samples alternative of the permutation test, which has the null hypothesis that the observations within each pair representing the same network before and after modulation is drawn from the same underlying distribution. To correct for the multiple comparisons that we made, we applied the Holm-Bonferroni correction. For the comparisons made between strategies (Fig 4) and the total inhibitory versus excitatory population (Fig 5a) we corrected for the 5 comparisons made on the same groups, whereas for the excitatory vs inhibitory comparisons (Fig 5b and 5c) we corrected for the 20 comparisons made. For the correlation between the number of selective units and the different metrics of behavior, we calculated the Spearman correlation coefficient with associated p-value. Raw and adjusted p-values can be found in S1 Table.

An important point to keep in mind when assesing the statistical comparisons in our study, and particularly the comparisons across neural selectivity groups and behavioral strategies, is that results of statistically significant differences have to be considered in the proper context of the available data sets. It is known that, for studies in which extremely large data sets are available, or in simulation studies where the size of the data set can be arbitrarily increased, statistically significant results may appear even for arbitrarily small and negligible effects [51]. We therefore restricted ourselves to an initial number of networks (N = 200) which provided a good estimate of the network-averaged psychometric curves, and analyzed this data set accordingly by following standard statistical guidelines.

## Supporting information

S1 Fig Decision threshold influence on classification accuracy and valid trials. a We explored the effect of different thresholds for the difference between the output variables that is used to mark a network’s decision time. We observed that the lower the threshold, the more errors the networks make and that the classification accuracy increases until a threshold of 0.3, after which it drops again, likely due to less decisions being made at higher thresholds. The candidate threshold range that achieved a classification accuracy over 80% were 0.2 – 0.35. **b** The lower the imposed threshold, the more decisions the model already made before the stimulus onset, which we marked as invalid trials. Especially thresholds below 0.2 result in a relatively large fraction of trials becoming invalid. **c** The number of trials in which no decision is made, so where the output variables of the networks do not separate enough, increases with the decision threshold. Especially thresholds larger than 0.25 result in a relatively large fraction of trials becoming invalid.
(TIFF)

S2 Fig Speed-accuracy trade-off. A positive correlation of 0.47 (p < 0.001, Pearson R) is apparent between the accuracy of networks and the mean reaction time, in line with earlier reports of a speed-accuracy trade-off reported in both animal and computational studies.
(TIFF)

S3 Fig Summary of results when psychometric functions are fitted with lapse parameters.**a** Example of a standard sigmoidal function fitted to data of a network as used in this research. **b** Example of the same data as in a but fitted with a sigmoidal function with lapse parameters. **c** $\gamma_{r}$) and low-choices at the highest frequency ($\gamma_{\lambda }$), changing the range of the psychometric function from (0, 1) to ($\gamma_{r},\;\text{1}-\;\gamma_{\lambda }$). d Similarly to Fig 3b, we observe spread in all metrics of behavior where high accuracy and low mean reaction times are mostly achieved in multisensory trials. **e** Similar to Fig 3c, 2D projections of **d** reveal that networks achieve low reaction times on multisensory trials (left) while still achieving high accuracy. The bias also seems to be reduced with a better accuracy (center) which is mostly achieved on multisensory trials. The bias level does not seem to change a lot with reaction times, but it is again apparent that most low reaction times are achieved on multisensory trials. **f** Distribution plots of e show again an average lower reaction time on multisensory trials as compared to unimodal trials (left), a wider spread in accuracy on multisensory trials (center) and a narrower distribution of bias levels on multisensory trials (right). The difference between multisensory and unimodal bias seems smaller compared to Fig 3d. **g** Fitting psychometric curves to the data obtained after application of a modulatory current reveals that often there is no significant effect on the accuracy (slope) directly, but that often there is a shift in lapse parameters that can account for the observed change in accuracy in the standard sigmoidal fitting. See section A of the S1 Table. **h** When looking at the difference between accurate and inaccurate networks based on the accuracy of networks after fitting with a sigmoidal function with lapse parameters, we see no significant changes in choice selective neurons anymore, as observed in Fig 4d. However, the slope of this sigmoid might not capture the accuracy as explicit anymore since the lapse parameters are not included in the definition of accuracy in this situation and a more elaborate definition of accuracy would be better suitable.(TIFF)

S4 Fig Summary of results for RNN not adhering to the Dale’s law. **a** Sigmoidal functions fitted to data from visual, auditory, or audiovisual trials. **b** Similarly to Fig 3b, we observe spread in all metrics of behavior where high accuracy and low reaction times are mostly achieved in multisensory trials. **c** Same as b, but displayed via multiple 2D projections. **d** Distribution plots of c showing different spreading profiles. **e** Number of selective units across all selectivity groups for fast vs slow networks (top panels) and accurate vs inaccurate networks (bottom panels).
(TIFF)

S5 Fig Inhibitory populations do not contribute to differences in behavioral strategies. **a** Fraction of inhibitory units across all selectivity groups for fast vs slow networks. **b** Same as a, but for accurate vs inaccurate networks. Significance: * p < 0.05, ** p < 0.01, *** p < 0.001 two-tailed permutation test with n = 100,000 resamples and Holm-Bonferroni correction.
(TIFF)

S6 Fig ROC analysis to mark selectivity results in mostly mixed-selective units. **a** Adding a modulatory current to selective units based on the ROC method results in no significant effects for choice selective units. The reason for this is likely because of the low number of neurons that are marked as pure-choice selective, as most neurons are marked to be mixed selective following the ROC approach. The large number of mixed selective units also results in a significant decrease in accuracy when these neurons are targeted as compared to Fig 6c where there was no significant effect visible after targeting mixed selective units, likely because there were less mixed selective units present. **b** There do not seem to be any significant differences between fast and slow groups using the ROC definition, likely because almost all neurons are marked to be mixed selective as compared to our rate-based classification approach. **C** Same as **b** but for accurate versus inaccurate networks.
(TIFF)

S7 Fig Lesioning silent neurons reveals functional role. **a** We lesioned neurons that were silent by forcing their firing rate to be zero at every timestep. **b** Comparing the networks’ performances before and after lesioning reveals that only the accuracy is significantly increased by lesioning the silent neurons. **c** Lesion of the choice and silent neurons improves reaction times, while lesioning silent or hyper-selective neurons improves accuracy. Mixed-selective neurons, however, lead to a decreased accuracy when lesioned.
(TIFF)

S1 Table Statistics for the model comparison (all figures). (DOCX)

## References

[1] Kanai R, Rees G. The structural basis of inter-individual differences in human behaviour and cognition. Nat Rev Neurosci. 2011;12(4):231–42. doi: 10.1038/nrn3000 21407245

[2] Faure P, Fayad SL, Solié C, Reynolds LM. Social Determinants of Inter-Individual Variability and Vulnerability: The Role of Dopamine. Front Behav Neurosci. 2022;16:836343. doi: 10.3389/fnbeh.2022.836343 35386723 PMC8979673

[3] Buchanan SM, Kain JS, de Bivort BL. Neuronal control of locomotor handedness in Drosophila. Proc Natl Acad Sci U S A. 2015;112(21):6700–5. doi: 10.1073/pnas.1500804112 25953337 PMC4450378

[4] Stern S, Kirst C, Bargmann CI. Neuromodulatory Control of Long-Term Behavioral Patterns and Individuality across Development. Cell. 2017;171(7):1649-1662.e10. doi: 10.1016/j.cell.2017.10.041 29198526

[5] Tuttle AH, Philip VM, Chesler EJ, Mogil JS. Comparing phenotypic variation between inbred and outbred mice. Nat Methods. 2018;15(12):994–6. doi: 10.1038/s41592-018-0224-7 30504873 PMC6518396

[6] Brunton BW, Botvinick MM, Brody CD. Rats and humans can optimally accumulate evidence for decision-making. Science. 2013;340(6128):95–8. doi: 10.1126/science.1233912 23559254

[7] Shadlen MN, Newsome WT. Neural basis of a perceptual decision in the parietal cortex (area LIP) of the rhesus monkey. J Neurophysiol. 2001;86(4):1916–36. doi: 10.1152/jn.2001.86.4.1916 11600651

[8] International Brain Laboratory, Aguillon-Rodriguez V, Angelaki D, Bayer H, Bonacchi N, Carandini M, et al. Standardized and reproducible measurement of decision-making in mice. Elife. 2021;10:e63711. doi: 10.7554/eLife.63711 34011433 PMC8137147

[9] Wang X-J. Probabilistic decision making by slow reverberation in cortical circuits. Neuron. 2002;36(5):955–68. doi: 10.1016/s0896-6273(02)01092-9 12467598

[10] Sheppard JP, Raposo D, Churchland AK. Dynamic weighting of multisensory stimuli shapes decision-making in rats and humans. J Vis. 2013;13(6):4. doi: 10.1167/13.6.4 23658374 PMC3649064

[11] Raposo D, Sheppard JP, Schrater PR, Churchland AK. Multisensory decision-making in rats and humans. J Neurosci. 2012;32(11):3726–35. doi: 10.1523/JNEUROSCI.4998-11.2012 22423093 PMC3335889

[12] Chandrasekaran C. Computational principles and models of multisensory integration. Curr Opin Neurobiol. 2017;43:25–34. doi: 10.1016/j.conb.2016.11.002 27918886 PMC5447489

[13] Angelaki DE, Gu Y, DeAngelis GC. Multisensory integration: psychophysics, neurophysiology, and computation. Curr Opin Neurobiol. 2009;19(4):452–8. doi: 10.1016/j.conb.2009.06.008 19616425 PMC2749464

[14] Meijer GT, Marchesi P, Mejias JF, Montijn JS, Lansink CS, Pennartz CMA. Neural Correlates of Multisensory Detection Behavior: Comparison of Primary and Higher-Order Visual Cortex. Cell Rep. 2020;31(6):107636. doi: 10.1016/j.celrep.2020.107636 32402272

[15] Oude Lohuis MN, Pie JL, Marchesi P, Montijn JS, de Kock CPJ, Pennartz CMA, et al. Multisensory task demands temporally extend the causal requirement for visual cortex in perception. Nat Commun. 2022;13(1):2864. doi: 10.1038/s41467-022-30600-4 35606448 PMC9126973

[16] Bechara A, Dolan S, Hindes A. Decision-making and addiction (part II): myopia for the future or hypersensitivity to reward?. Neuropsychologia. 2002;40(10):1690–705. doi: 10.1016/s0028-3932(02)00016-7 11992657

[17] Pittaras E, Hamelin H, Granon S. Inter-Individual Differences in Cognitive Tasks: Focusing on the Shaping of Decision-Making Strategies. Front Behav Neurosci. 2022;16:818746. doi: 10.3389/fnbeh.2022.818746 35431831 PMC9007591

[18] Pagan M, Tang VD, Aoi MC, Pillow JW, Mante V, Sussillo D, et al. A new theoretical framework jointly explains behavioral and neural variability across subjects performing flexible decision-making. bioRxiv; 2022. p. 2022.11.28.518207. 10.1101/2022.11.28.518207

[19] Ashwood ZC, Roy NA, Stone IR, International Brain Laboratory, Urai AE, Churchland AK, et al. Mice alternate between discrete strategies during perceptual decision-making. Nat Neurosci. 2022;25(2):201–12. doi: 10.1038/s41593-021-01007-z 35132235 PMC8890994

[20] Le NM, Yildirim M, Wang Y, Sugihara H, Jazayeri M, Sur M. Mixtures of strategies underlie rodent behavior during reversal learning. PLoS Comput Biol. 2023;19(9):e1011430. doi: 10.1371/journal.pcbi.1011430 37708113 PMC10501641

[21] Marsat G, Maler L. Neural heterogeneity and efficient population codes for communication signals. J Neurophysiol. 2010;104(5):2543–55. doi: 10.1152/jn.00256.2010 20631220

[22] Mejias JF, Longtin A. Optimal heterogeneity for coding in spiking neural networks. Phys Rev Lett. 2012;108(22):228102. doi: 10.1103/PhysRevLett.108.228102 23003656

[23] Mejias JF, Longtin A. Differential effects of excitatory and inhibitory heterogeneity on the gain and asynchronous state of sparse cortical networks. Frontiers in Computational Neuroscience. 2014;8:107. Available: https://www.frontiersin.org/articles/10.3389/fncom.2014.00107 25309409 10.3389/fncom.2014.00107 PMC4162374

[24] Zeldenrust F, Gutkin B, Denéve S. Efficient and robust coding in heterogeneous recurrent networks. PLoS Comput Biol. 2021;17(4):e1008673. doi: 10.1371/journal.pcbi.1008673 33930016 PMC8115785

[25] Perez-Nieves N, Leung VCH, Dragotti PL, Goodman DFM. Neural heterogeneity promotes robust learning. Nat Commun. 2021;12(1):5791. doi: 10.1038/s41467-021-26022-3 34608134 PMC8490404

[26] Mante V, Sussillo D, Shenoy KV, Newsome WT. Context-dependent computation by recurrent dynamics in prefrontal cortex. Nature. 2013;503(7474):78–84. doi: 10.1038/nature12742 24201281 PMC4121670

[27] Sussillo D, Barak O. Opening the black box: low-dimensional dynamics in high-dimensional recurrent neural networks. Neural Comput. 2013;25(3):626–49. doi: 10.1162/NECO_a_00409 23272922

[28] Song HF, Yang GR, Wang X-J. Training Excitatory-Inhibitory Recurrent Neural Networks for Cognitive Tasks: A Simple and Flexible Framework. PLoS Comput Biol. 2016;12(2):e1004792. doi: 10.1371/journal.pcbi.1004792 26928718 PMC4771709

[29] Yang GR, Joglekar MR, Song HF, Newsome WT, Wang X-J. Task representations in neural networks trained to perform many cognitive tasks. Nat Neurosci. 2019;22(2):297–306. doi: 10.1038/s41593-018-0310-2 30643294 PMC11549734

[30] Yang GR, Wang X-J. Artificial Neural Networks for Neuroscientists: A Primer. Neuron. 2020;107(6):1048–70. doi: 10.1016/j.neuron.2020.09.005 32970997 PMC11576090

[31] Kleinman M, Chandrasekaran C, Kao J. A mechanistic multi-area recurrent network model of decision-making. In: Ranzato M, Beygelzimer A, Dauphin Y, Liang PS, Vaughan JW, Editors. Advances in Neural Information Processing Systems. Curran Associates, Inc.; 2021. pp. 23152–65. Available: https://proceedings.neurips.cc/paper_files/paper/2021/file/c2f599841f21aaefeeabd2a60ef7bfe8-Paper.pdf

[32] Yang GR, Mazon MM. Next-generation of recurrent neural network models for cognition. PsyArXiv; 2021. doi: 10.31234/osf.io/w34n2

[33] Raposo D, Kaufman MT, Churchland AK. A category-free neural population supports evolving demands during decision-making. Nat Neurosci. 2014;17(12):1784–92. doi: 10.1038/nn.3865 25383902 PMC4294797

[34] Rigotti M, Barak O, Warden MR, Wang X-J, Daw ND, Miller EK, et al. The importance of mixed selectivity in complex cognitive tasks. Nature. 2013;497(7451):585–90. doi: 10.1038/nature12160 23685452 PMC4412347

[35] Roach JP, Churchland AK, Engel TA. Choice selective inhibition drives stability and competition in decision circuits. Nat Commun. 2023;14(1):147. doi: 10.1038/s41467-023-35822-8 36627310 PMC9832138

[36] Heitz RP, Schall JD. Neural mechanisms of speed-accuracy tradeoff. Neuron. 2012;76(3):616–28. doi: 10.1016/j.neuron.2012.08.030 23141072 PMC3576837

[37] Heitz RP. The speed-accuracy tradeoff: history, physiology, methodology, and behavior. Front Neurosci. 2014;8:150. doi: 10.3389/fnins.2014.00150 24966810 PMC4052662

[38] Standage D, Blohm G, Dorris MC. On the neural implementation of the speed-accuracy trade-off. Front Neurosci. 2014;8:236. doi: 10.3389/fnins.2014.00236 25165430 PMC4131279

[39] Alais D, Newell FN, Mamassian P. Multisensory processing in review: from physiology to behaviour. Seeing Perceiving. 2010;23(1):3–38. doi: 10.1163/187847510X488603 20507725

[40] Meijer GT, Pie JL, Dolman TL, Pennartz CMA, Lansink CS. Audiovisual Integration Enhances Stimulus Detection Performance in Mice. Front Behav Neurosci. 2018;12:231. doi: 10.3389/fnbeh.2018.00231 30337861 PMC6180166

[41] Meijer GT, Mertens PEC, Pennartz CMA, Olcese U, Lansink CS. The circuit architecture of cortical multisensory processing: Distinct functions jointly operating within a common anatomical network. Prog Neurobiol. 2019;174:1–15. doi: 10.1016/j.pneurobio.2019.01.004 30677428

[42] Wong K-F, Wang X-J. A recurrent network mechanism of time integration in perceptual decisions. J Neurosci. 2006;26(4):1314–28. doi: 10.1523/JNEUROSCI.3733-05.2006 16436619 PMC6674568

[43] Moreni G, Dorcioman RA, Pennartz CMA, Mejias JF. Cell-type-specific firing patterns in a V1 cortical column model depend on feedforward and feedback-driven states. PLoS Comput Biol. 2025;21(4):e1012036. doi: 10.1371/journal.pcbi.1012036 40267074 PMC12017539

[44] Moreni G, Zou L, Pennartz CMA, Mejias JF. Synaptic plasticity facilitates oscillations in a V1 cortical column model with multiple interneuron types. Front Comput Neurosci. 2025;19:1568143. doi: 10.3389/fncom.2025.1568143 40370493 PMC12075120

[45] Najafi F, Elsayed GF, Cao R, Pnevmatikakis E, Latham PE, Cunningham JP, et al. Excitatory and Inhibitory Subnetworks Are Equally Selective during Decision-Making and Emerge Simultaneously during Learning. Neuron. 2020;105(1):165-179.e8. doi: 10.1016/j.neuron.2019.09.045 31753580 PMC6952547

[46] Murray JD, Jaramillo J, Wang X-J. Working Memory and Decision-Making in a Frontoparietal Circuit Model. J Neurosci. 2017;37(50):12167–86. doi: 10.1523/JNEUROSCI.0343-17.2017 29114071 PMC5729190

[47] Mejías JF, Wang X-J. Mechanisms of distributed working memory in a large-scale network of macaque neocortex. Elife. 2022;11:e72136. doi: 10.7554/eLife.72136 35200137 PMC8871396

[48] Jaramillo J, Mejias JF, Wang X-J. Engagement of Pulvino-cortical Feedforward and Feedback Pathways in Cognitive Computations. Neuron. 2019;101(2):321-336.e9. doi: 10.1016/j.neuron.2018.11.023 30553546 PMC6650151

[49] Lindeman S, Hong S, Kros L, Mejias JF, Romano V, Oostenveld R, et al. Cerebellar Purkinje cells can differentially modulate coherence between sensory and motor cortex depending on region and behavior. Proc Natl Acad Sci U S A. 2021;118(2):e2015292118. doi: 10.1073/pnas.2015292118 33443203 PMC7812746

[50] Prins N. The psychometric function: the lapse rate revisited. J Vis. 2012;12(6):25. doi: 10.1167/12.6.25 22715196

[51] Lin M, Lucas HC Jr, Shmueli G. Research Commentary—Too Big to Fail: Large Samples and the p-Value Problem. Information Systems Research. 2013;24(4):906–17. doi: 10.1287/isre.2013.0480

