# Supplementary figures and images for: Diverse and flexible behavioral strategies arise in recurrent neural networks trained on multisensory decision making
Source: PLoS Comput Biol. 2025 Oct 9;21(10):e1013559. doi: 10.1371/journal.pcbi.1013559 (PMC12520346; doi:10.1371/journal.pcbi.1013559)

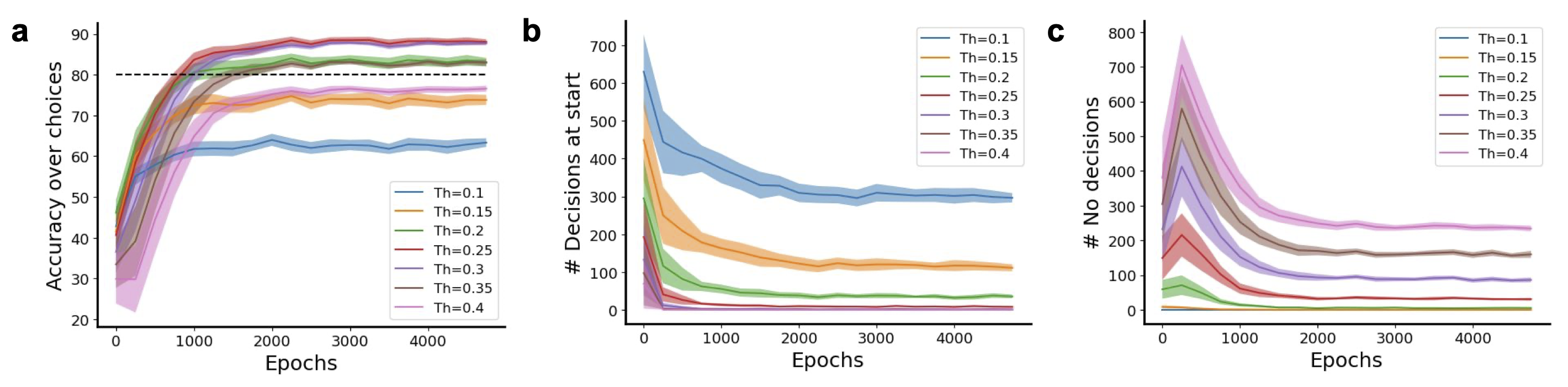

Supplement: S1 Fig — a We explored the effect of different thresholds for the difference between the output variables that is used to mark a network’s decision time. We observed that the lower the threshold, the more errors the networks make and that the classification accuracy increases until a threshold of 0.3, after which it drops again, likely due to less decisions being made at higher thresholds. The candidate threshold range that achieved a classification accuracy over 80% were 0.2 – 0.35. b The lower the imposed threshold, the more decisions the model already made before the stimulus onset, which we marked as invalid trials. Especially thresholds below 0.2 result in a relatively large fraction of trials becoming invalid. c The number of trials in which no decision is made, so where the output variables of the networks do not separate enough, increases with the decision threshold. Especially thresholds larger than 0.25 result in a relatively large fraction of trials becoming invalid. (TIFF) [file pcbi.1013559.s001.tiff]

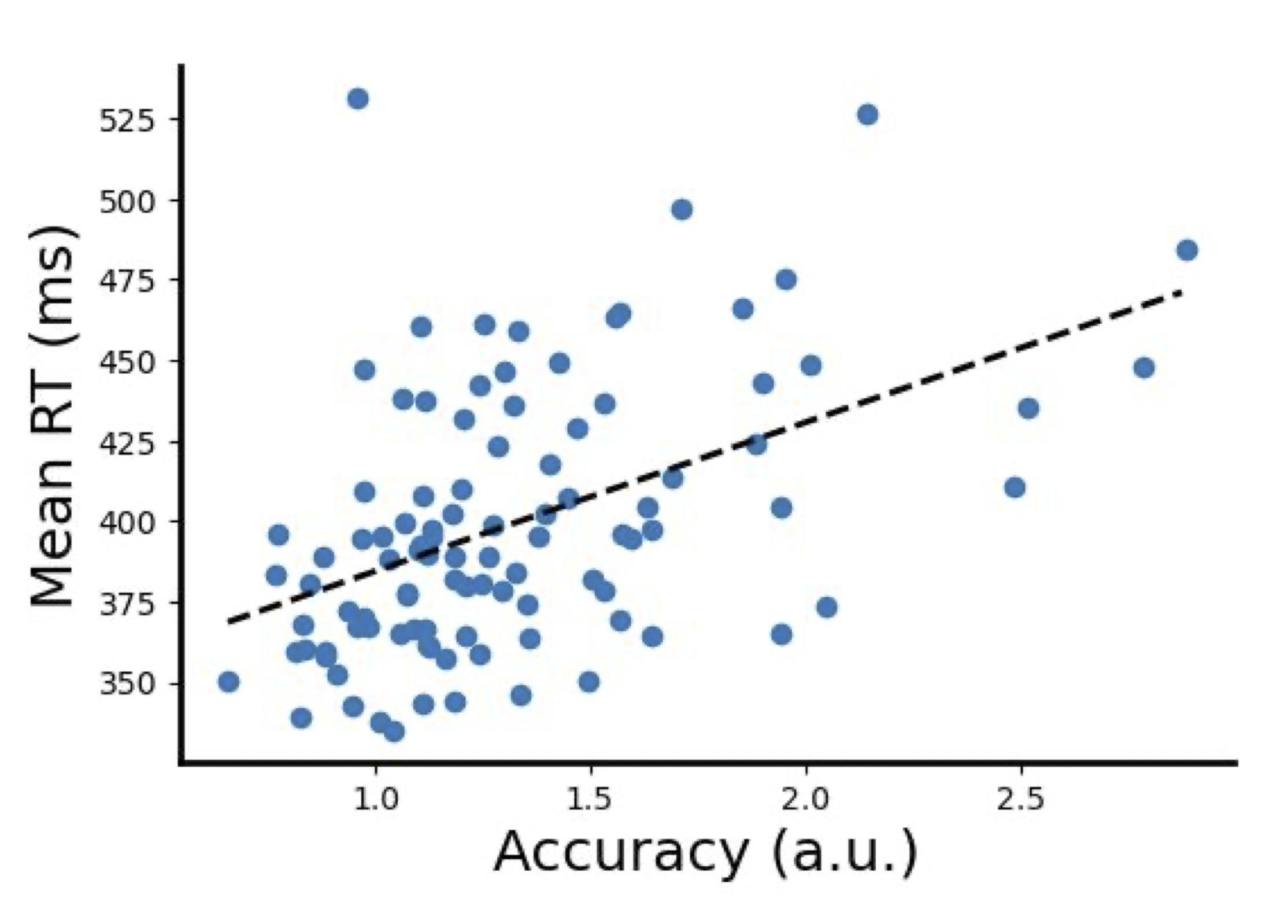

Supplement: S2 Fig — A positive correlation of 0.47 (p < 0.001, Pearson R) is apparent between the accuracy of networks and the mean reaction time, in line with earlier reports of a speed-accuracy trade-off reported in both animal and computational studies. (TIFF) [file pcbi.1013559.s002.tiff]

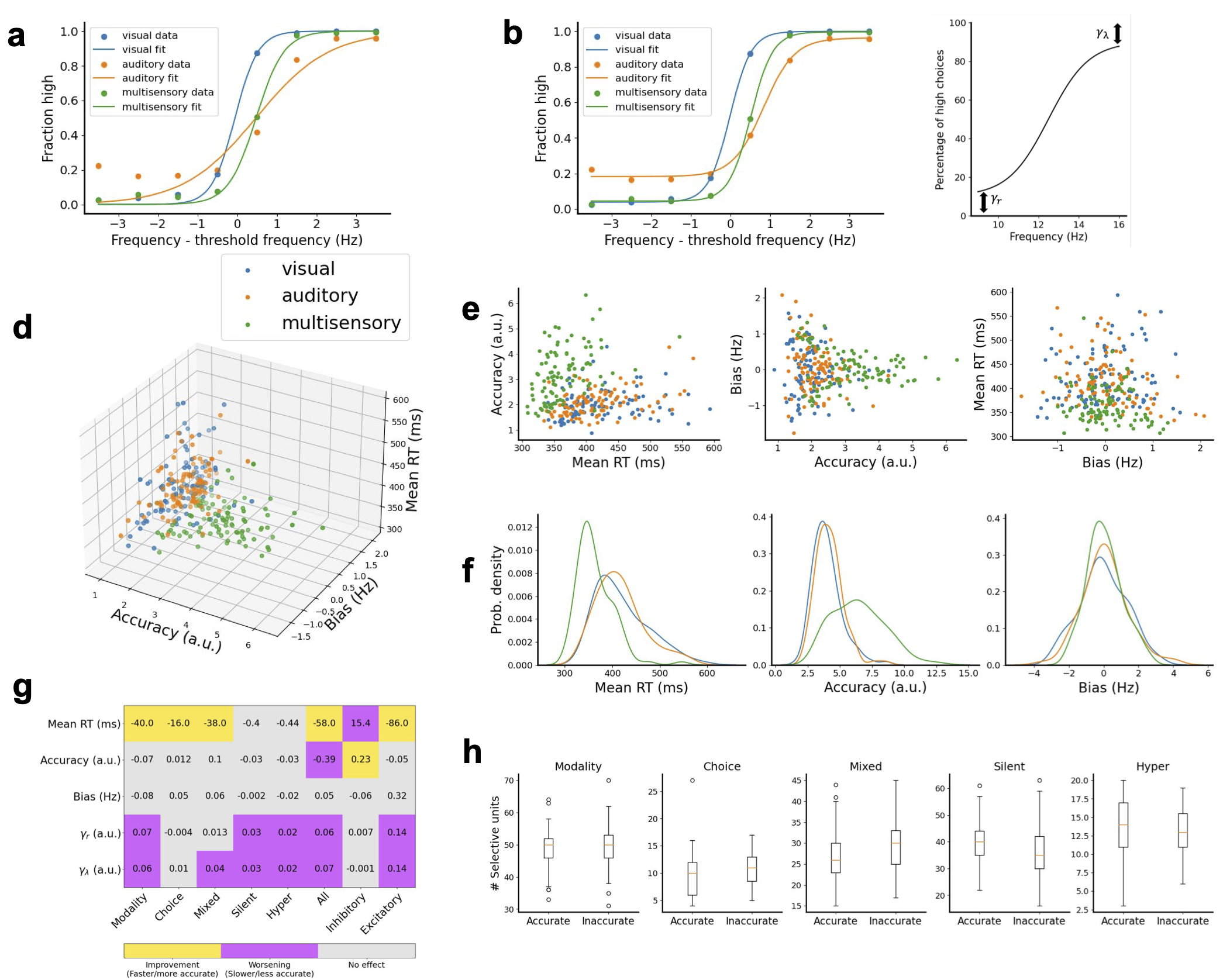

Supplement: S3 Fig — a Example of a standard sigmoidal function fitted to data of a network as used in this research. b Example of the same data as in a but fitted with a sigmoidal function with lapse parameters. c γr) and low-choices at the highest frequency (γλ), changing the range of the psychometric function from (0, 1) to (γr, 1− γλ). d Similarly to Fig 3b, we observe spread in all metrics of behavior where high accuracy and low mean reaction times are mostly achieved in multisensory trials. e Similar to Fig 3c, 2D projections of d reveal that networks achieve low reaction times on multisensory trials (left) while still achieving high accuracy. The bias also seems to be reduced with a better accuracy (center) which is mostly achieved on multisensory trials. The bias level does not seem to change a lot with reaction times, but it is again apparent that most low reaction times are achieved on multisensory trials. f Distribution plots of e show again an average lower reaction time on multisensory trials as compared to unimodal trials (left), a wider spread in accuracy on multisensory trials (center) and a narrower distribution of bias levels on multisensory trials (right). The difference between multisensory and unimodal bias seems smaller compared to Fig 3d. g Fitting psychometric curves to the data obtained after application of a modulatory current reveals that often there is no significant effect on the accuracy (slope) directly, but that often there is a shift in lapse parameters that can account for the observed change in accuracy in the standard sigmoidal fitting. See section A of the S1 Table. h When looking at the difference between accurate and inaccurate networks based on the accuracy of networks after fitting with a sigmoidal function with lapse parameters, we see no significant changes in choice selective neurons anymore, as observed in Fig 4d. However, the slope of this sigmoid might not capture the accuracy as explicit anymore since the lapse parameters are not included [file pcbi.1013559.s003.tiff]

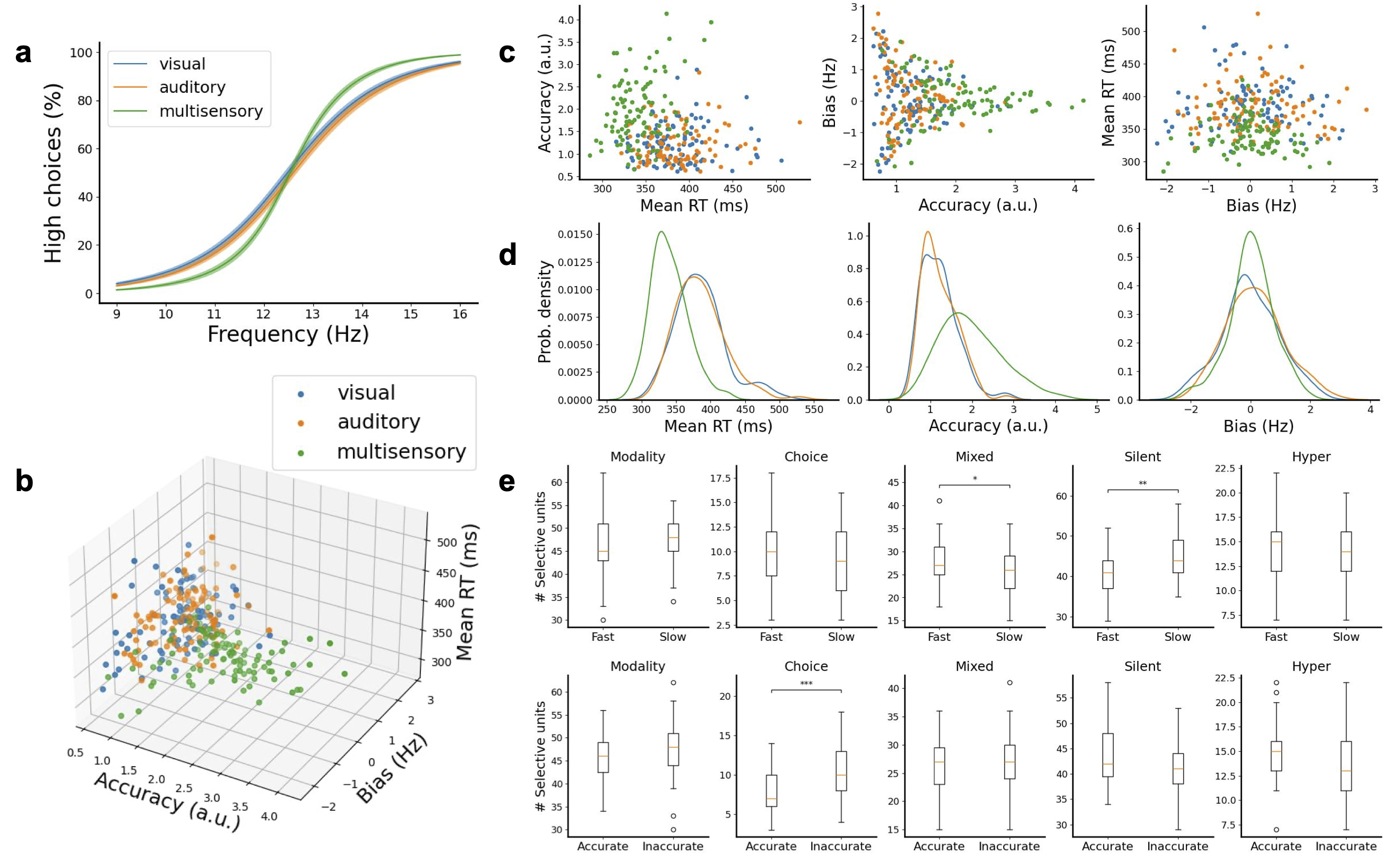

Supplement: S4 Fig — a Sigmoidal functions fitted to data from visual, auditory, or audiovisual trials. b Similarly to Fig 3b, we observe spread in all metrics of behavior where high accuracy and low reaction times are mostly achieved in multisensory trials. c Same as b, but displayed via multiple 2D projections. d Distribution plots of c showing different spreading profiles. e Number of selective units across all selectivity groups for fast vs slow networks (top panels) and accurate vs inaccurate networks (bottom panels). (TIFF) [file pcbi.1013559.s004.tiff]

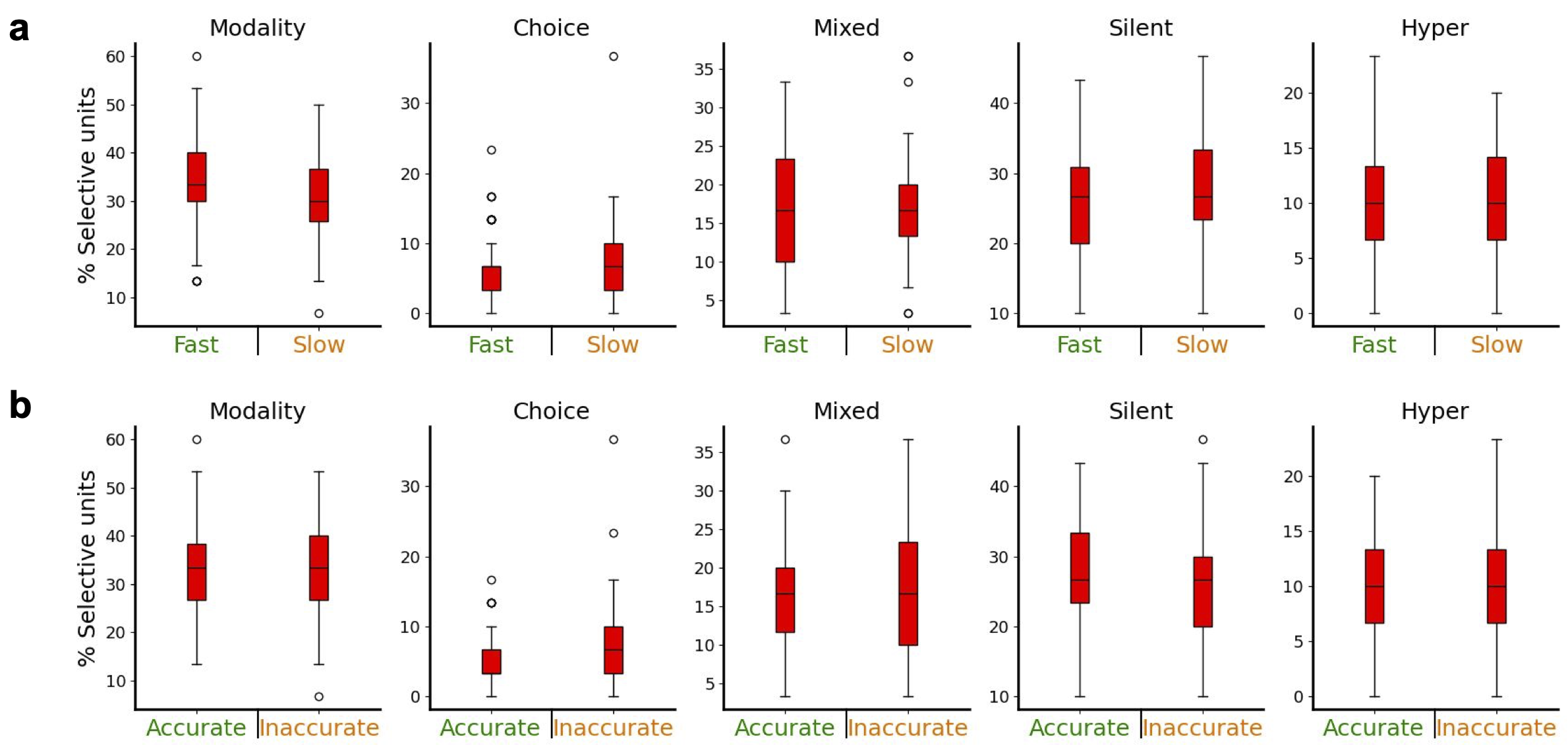

Supplement: S5 Fig — a Fraction of inhibitory units across all selectivity groups for fast vs slow networks. b Same as a, but for accurate vs inaccurate networks. Significance: * p < 0.05, ** p < 0.01, *** p < 0.001 two-tailed permutation test with n = 100,000 resamples and Holm-Bonferroni correction. (TIFF) [file pcbi.1013559.s005.tiff]

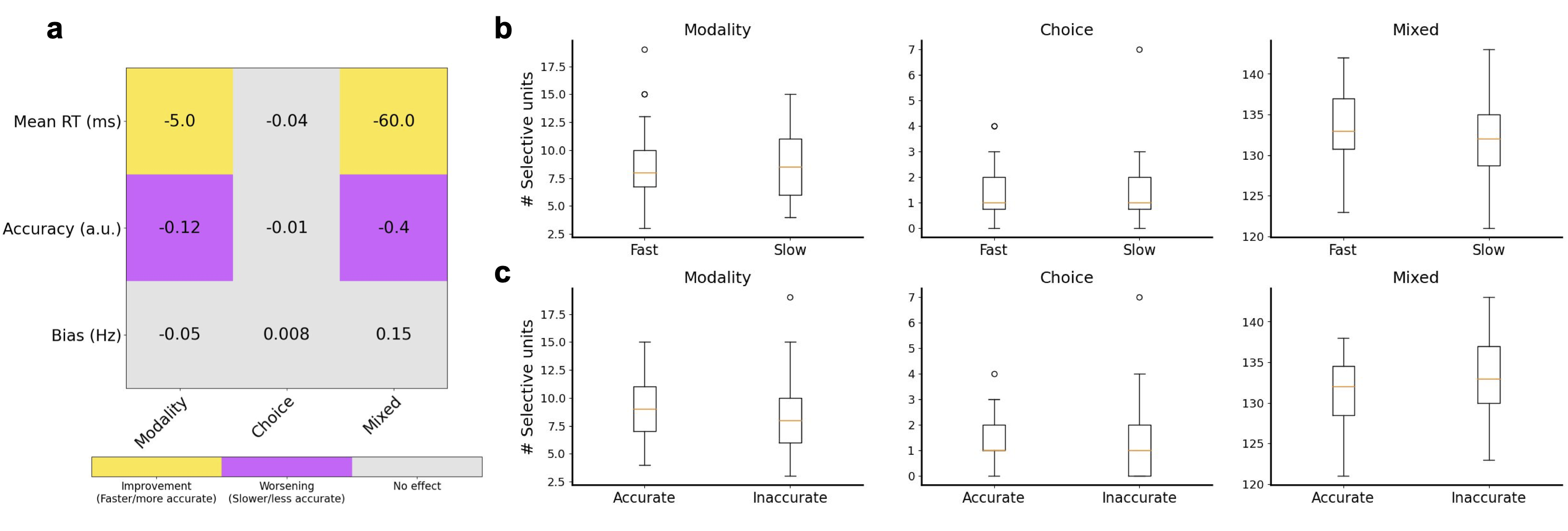

Supplement: S6 Fig — a Adding a modulatory current to selective units based on the ROC method results in no significant effects for choice selective units. The reason for this is likely because of the low number of neurons that are marked as pure-choice selective, as most neurons are marked to be mixed selective following the ROC approach. The large number of mixed selective units also results in a significant decrease in accuracy when these neurons are targeted as compared to Fig 6c where there was no significant effect visible after targeting mixed selective units, likely because there were less mixed selective units present. b There do not seem to be any significant differences between fast and slow groups using the ROC definition, likely because almost all neurons are marked to be mixed selective as compared to our rate-based classification approach. C Same as b but for accurate versus inaccurate networks. (TIFF) [file pcbi.1013559.s006.tiff]

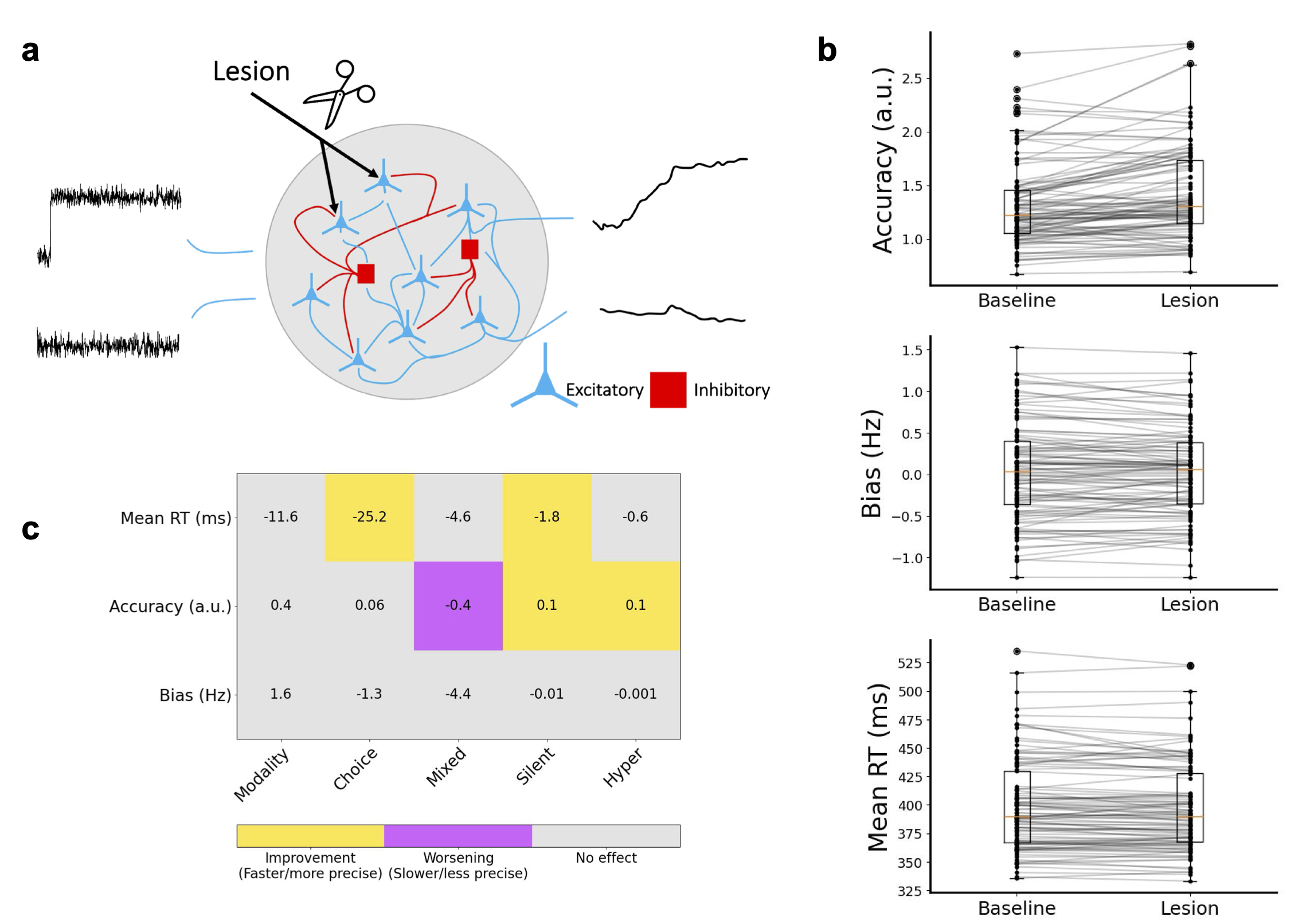

Supplement: S7 Fig — a We lesioned neurons that were silent by forcing their firing rate to be zero at every timestep. b Comparing the networks’ performances before and after lesioning reveals that only the accuracy is significantly increased by lesioning the silent neurons. c Lesion of the choice and silent neurons improves reaction times, while lesioning silent or hyper-selective neurons improves accuracy. Mixed-selective neurons, however, lead to a decreased accuracy when lesioned. (TIFF) [file pcbi.1013559.s007.tiff]
