# Supplementary material for: Diverse and flexible behavioral strategies arise in recurrent neural networks trained on multisensory decision making
Source: PLoS Comput Biol. 2025 Oct 9;21(10):e1013559. doi: 10.1371/journal.pcbi.1013559 (PMC12520346; doi:10.1371/journal.pcbi.1013559)
Supplement: S1 Table — (DOCX) [file pcbi.1013559.s008.docx]

**Supplementary Table S1**

Section A: Raw p-values based on the permutation test for the application of a modulatory current as compared to baseline performance with lapse fitting (Fig. S3g).

|  | Slope | Offset | Mean RT | $\gamma_{r}$ | $\gamma_{\lambda}$ |
| --- | --- | --- | --- | --- | --- |
| Modality | 0.803871961 | 0.35097649 | 1.99998E-05 | 1.99998E-05 | 1.99998E-05 |
| Choice | 0.783672163 | 0.544334557 | 1.99998E-05 | 0.644593554 | 0.288117119 |
| Mixed | 0.865191348 | 0.618733813 | 1.99998E-05 | 0.281637184 | 0.008779912 |
| Silent | 0.400435996 | 0.925330747 | 0.638213618 | 1.99998E-05 | 1.99998E-05 |
| Hyper | 0.445255547 | 0.30501695 | 0.60601394 | 0.000159998 | 1.99998E-05 |
| All | 1.99998E-05 | 0.696353036 | 1.99998E-05 | 0.000259997 | 1.99998E-05 |
| Inhibitory | 1.99998E-05 | 0.430095699 | 1.99998E-05 | 0.246197538 | 0.876691233 |
| Excitatory | 0.909610904 | 0.364396356 | 1.99998E-05 | 1.99998E-05 | 1.99998E-05 |

Section B: Raw and adjusted p-values based on the permutation test and Holm-Bonferroni correction for difference in strategies (Fig 4c and d).

|  | Fast versus slow | | Accurate versus inaccurate | |
| --- | --- | --- | --- | --- |
|  | *p-value* | *Adjusted p-value* | *p-value* | *Adjusted p-value* |
| Modality | 0.0139199 | 2.63197368e-02 | 0.5354546 | 0.5354546 |
| Choice | 0.0149199 | 2.63197368e-02 | 0.000359996 | 0.00179998 |
| Mixed | 3.99998e-05 | 1.59998400e-04 | 0.00335996 | 0.01343987 |
| Silent | 1.99998e-05 | 9.99990000e-05 | 0.0087799 | 0.02633974 |
| Hyper | 0.00211998 | 5.81994180e-03 | 0.1263187 | 0.25263747 |

Section C: Correlations between the number of selective units and metrics of behavior (Spearman’s rho).

|  | Slope | Offset | Mean RT |
| --- | --- | --- | --- |
| Modality | -0.05 (p: 0.6) | -0.063 (p: 0.53) | -0.21 (p: 0.034) |
| Choice | -0.3 (p: < 0.01) | 0.1 (p: 0.32) | -0.42 (p: < 0.001) |
| Mixed | -0.29 (p: < 0.01) | 0.038 (p: 0.7) | -0.45 (p < 0.001) |
| Silent | 0.36 (p: < 0.001) | -0.07(p: 0.49) | 0.49 (p: < 0.001) |
| Hyper | 0.18 (p: 0.08) | -0.026 (p: 0.80) | 0.32 (p: < 0.01) |

Section D: Raw and adjusted p-values based on the permutation test and Holm-Bonferroni correction for differences in strategies in network not abiding to the Dale’s law (Fig. S4).

|  | *Fast versus slow* | | *Accurate versus inaccurate* | |
| --- | --- | --- | --- | --- |
|  | *p-value* | *Adjusted p-value* | *p-value* | *Adjusted p-value* |
| *Modality* | 0.4886151 | 0.97723023 | 0.27531724 | 0.55063449 |
| *Choice* | 0.1674783 | 0.50243498 | 0.0001200 | 0.00059999 |
| *Mixed* | 0.0066799 | 0.02671973 | 0.4441555 | 0.55063449 |
| *Silent* | 0.00036000 | 0.00179998 | 0.0342197 | 0.13687863 |
| *Hyper* | 0.6451735 | 0.97723023 | 0.0362396 | 0.13687863 |

Section E: Raw and adjusted p-values based on the permutation test and Holm-Bonferroni correction for difference in EI (Fig. 5a).

|  | Inhibitory versus Excitatory | |
| --- | --- | --- |
|  | *p-value* | *Adjusted p-value* |
| Modality | 5.76654233e-01 | 5.82634174e-01 |
| Choice | 4.47995520e-03 | 8.47991520e-03 |
| Mixed | 1.99998000e-05 | 9.99990000e-05 |
| Silent | 2.15997840e-03 | 5.75994240e-03 |
| Hyper | 1.99998000e-05 | 9.99990000e-05 |

Section F: Raw and adjusted p-values based on the permutation test and Holm-Bonferroni correction for difference in strategies between EI related to speed (Fig. 5b and Fig. S5).

|  | *Exc. Fast v Exc. Slow* | | *Exc. Accurate v Exc. Inaccurate* | |
| --- | --- | --- | --- | --- |
|  | *p-value* | *Adjusted p-value* | *p-value* | *Adjusted p-value* |
| *Modality* | 0.063399 | 0.063399 | 0.686833 | 0.686833 |
| *Choice* | 0.000860 | 0.002580 | 0.000120 | 0.000600 |
| *Mixed* | 0.000020 | 0.000100 | 0.005360 | 0.021440 |
| *Silent* | 0.000020 | 0.000100 | 0.006880 | 0.021440 |
| *Hyper* | 0.026560 | 0.053119 | 0.209898 | 0.419796 |

|  | *Inh. Fast v Inh. Slow* | | *Inh. Accurate v Inh. Inaccurate* | |
| --- | --- | --- | --- | --- |
|  | *p-value* | *Adjusted p-value* | *p-value* | *Adjusted p-value* |
| *Modality* | 0.067659 | 0.270637 | 0.542855 | 1 |
| *Choice* | 0.420656 | 1 | 0.212098 | 1 |
| *Mixed* | 0.673613 | 1 | 0.331577 | 1 |
| *Silent* | 0.442236 | 1 | 0.491775 | 1 |
| *Hyper* | 0.017620 | 0.088099 | 0.376156 | 1 |

Section G: Raw p-values based on the permutation test for the application of a modulatory current as compared to baseline performance (Fig. 6c).

|  | *Slope* | *Offset* | *Mean RT* |
| --- | --- | --- | --- |
| *Modality* | 0.00002 | 0.261237 | 0.000020 |
| *Choice* | 0.03172 | 0.074199 | 0.000020 |
| *Mixed* | 0.04514 | 0.361836 | 0.000020 |
| *Silent* | 0.00002 | 0.797772 | 0.091499 |
| *Hyper* | 0.00002 | 0.706013 | 0.667813 |
| *All* | 0.00002 | 0.294197 | 0.000020 |
| *Inhibitory* | 0.00002 | 0.176738 | 0.000020 |
| *Excitatory* | 0.00002 | 0.656153 | 0.000020 |

Section H: Raw p-values based on the permutation test for the application of a modulatory current as compared to baseline performance using the ROC definition (Fig. S6a).

|  | Slope | Offset | Mean RT |
| --- | --- | --- | --- |
| Modality | 0.000020 | 0.249998 | 0.000020 |
| Choice | 0.552194 | 0.431536 | 0.939611 |
| Mixed | 0.000020 | 0.481515 | 0.000020 |

Section I: Raw p-values based on the permutation test for lesioning as compared to baseline performance (Fig. S5c).

|  | *Slope* | *Offset* | *Mean RT* |
| --- | --- | --- | --- |
| *Modality* | 0.086879 | 0.380316 | 0.377556 |
| *Choice* | 0.401816 | 0.359236 | 0.014600 |
| *Mixed* | 0.052059 | 0.098999 | 0.708113 |
| *Silent* | 0.000020 | 0.407556 | 0.018620 |
| *Hyper* | 0.000020 | 0.961810 | 0.469835 |
